# Spatial heterogeneity in discontinuation of modern spacing method in districts of India

**DOI:** 10.1186/s12978-021-01185-w

**Published:** 2021-06-30

**Authors:** Soumya Ranjan Nayak, Sanjay K. Mohanty, Bidhubhusan Mahapatra, Umakanta Sahoo

**Affiliations:** 1 Model Rural Health Research Unit, RMRCBB (ICMR), Tigiria, Cuttack, Odisha India; 2 International Institute for Population Sciences, Govandi Station Road, Mumbai, Maharashtra 400088 India; 3 Population Council of India, New Delhi, India

**Keywords:** Contraceptive, Modern spacing method, Discontinuation, India

## Abstract

**Background:**

Despite six decades of official family planning programme, the use of modern contraceptive method remained low in India. The discontinuation of modern spacing method (DMSM) has also increased from 42.3% in 2005−06 to 43.6% during 2015–16. Discontinuation rate is higher for Injectable (51%), followed by condom (47%), pill (42%) and lowest in IUD (26%).

**Methods:**

Data from NFHS-4 (2015–16) comprising of 601,509 households, 699,686 women and a sample of 119,548 episode of modern spacing method was used for the analysis. Multiple decrement life table has used to estimate 12-month discontinuation rate of modern spacing methods (DMSM). Moran’s I statistics, Bivariate LISA cluster map has used to understand the spatial correlates and clustering the DMSM. OLS model and impact analysis has used to assess the significant associated covariates with discontinuation.

**Result:**

The 12-month DMSM in India is 43.5%; largely due to desire for becoming pregnant and method failure. The high discontinuation rate was observed in most of the southern (62%) and central (46%) regions of India. DMSM has significantly and spatially associated with neighbouring districts of India (Moran’s I = 0.47, p-value = 0.00). The prevalence of modern spacing method is negatively associated with discontinuation in the neighbouring districts of India. The unmet need (β = 0.84, 95% CI 0.55–1.14), desire of children (β = 0.26, 95% CI − 0.05–0.57) and female sterilization (β = 0.54, 95% CI 0.14–0.95) were three main contributing factor to DMSM.

**Conclusion:**

Districts of high DMSM need programmatic intervention. More attention for counselling to client, health worker outreach to user and better quality care services will stimulate non-user of contraception.

**Supplementary Information:**

The online version contains supplementary material available at 10.1186/s12978-021-01185-w.

## Plain language summary

Contraceptive discontinuation is major issue of family planning of India. Without getting proper knowledge of modern spacing method, women facing serious health related issues so that they tend to discontinue their family planning method. We examine the spatial heterogeneity of discontinuation of modern spacing method in 640 district of India. Using secondary data of NFHS-4 (2015–16), we calculated 12-month discontinuation rate of any method and any modern spacing method by reason of discontinuation, also we presented discontinuation pattern by regions of India. To measure the spill over effect and associated factor of discontinuation of modern spacing method, we used OLS model and estimated Impact result. The findings of our study conveys that desire to become pregnant is the leading cause of discontinuation for both any method and any modern spacing method (12.43%), followed by other fertility related reasons and methods relates reason’s (4.40%). Method failure, side effect of method and method related reason together accounts 12% of contraceptive discontinuation in India. Very low use of modern spacing method of districts should be given more attention for policy maker and planner to increase the use of modern spacing method. The districts of high discontinuation of modern spacing method need programmatic intervention. More attention for counselling to client, health worker outreach to user and better quality care services will stimulate non-user of contraception.

**Supplementary Information:**

The online version contains supplementary material available at 10.1186/s12978-021-01185-w.

## Introduction

Since International conference of population and development (ICPD), Cairo, 1994, access to increase the use of contraception and maternal care has been integrated in global and national development agenda. The MDGs and SDGs has explicitly included access to reproductive health services in health related goals [1]. Though countries are converging on use of contraception and maternal care services, but they differ widely with respect to use of methods, access to contraception, quality of care, unmet need and method choice [2, 3]. In developing countries, two-third of unintended birth caused by non-use of contraception and one-third unintended birth are due to contraceptive discontinuation [4, 5]. Use of contraception saves lives of mother and children by reducing high risk pregnancy, maternal and child mortality, under-nutrition among children, increases child schooling and improves economic well-being of households [6, 7].

Increasing contraceptive discontinuation is a concern to many national government and policy makers in developing countries. In almost every country contraceptive failure within a year of method use, higher among younger women [2, 8–12]. The low use of modern spacing method and the likelihood of discontinuation is higher among women with high unmet need for the family planning. Unintended pregnancy which lead to health problem mother and baby, financial stress and unsafe abortion are largely due to contraceptive discontinuation and method failure [13–16]. Reason of discontinuation varies by contraceptive method and broadly categorized as method failure, method related attributes, fertility related (desire for children), opposition from family, side effects, son preferences, partner’s disapproval and depend on number of sexual partner, type of partner and sexual experience [17–19]. Contraceptive discontinuation can be reduced by expanding choice of methods, information to clients, technical competence, interpersonal relations, follow-up and continuity mechanisms, and the appropriate constellation of services, which are component of overall quality of care [9].

India is the second most populous country in the world with 17.5% of the world population. Though the country is close to replacement level of fertility (TFR 2.2) in 2017, but great variation found across the country and half of the women use modern contraceptive method. The use of modern spacing method remained low, and skewed despite increase in comprehensive knowledge of contraception and availability of multiple contraceptive method. Though there has been an increasing trend of traditional method with low prevalence of modern spacing method as a result the discontinuation rate of spacing method is very high, about one-third women who use spacing method, discontinue before 12-month of use [10]. The unmet need for spacing is moderate and remained unchanged since 2005 [10]. Studies have focused on trends, differential, and determinants of contraceptive use [2, 11, 12, 20], but there are limited number of studies on discontinuation of contraception in India. Traditional contraceptive method has higher probability of method failure and discontinuation whereas in poor wealth quantile person, less educated person has more likelihood of discontinuation lead to unwanted birth [21]. The 12-month discontinuation rate is highest for condom followed by pill, injectable, IUD and lowest is implant in India [10].

Indian districts are heterogeneous in fertility level, contraceptive use and level of development [22]. The national and state average conceals large variation in contraceptive use and discontinuation across districts of India. The increasing use of traditional methods, high unmet need for modern spacing method, high unwanted childbearing, and low birth spacing necessitates an investigation of the correlates on DMSM in districts of India. No attempt has been made on understanding the spatial pattern of contraceptive discontinuation in districts of India. In this context the aim of this paper is to understand the spatial pattern of discontinuation and correlates of modern spacing method in India.

### Data and methods

#### Data

This paper based on the unit level data of fourth round of National family health survey NFHS-4, 2015–16 conducted by the International Institute for Population sciences under the aegis of *Ministry of Health and Family Welfare, Govt. of India*. NFHS-4 was a nationwide survey, that had successfully interviewed 601,509 households, 699,686 women in the reproductive age group of 15–49 years and 112,122 men aged 15–54 years. The survey collected comprehensive information on demographic, socioeconomic, contraception, nutrition and many other information relating to mother and children. Detail history of contraceptive use was. collected using the “calendar” method that record monthly history of contraceptive methods, births and pregnancies in 60-month period prior to survey. For the analysis purpose event/episode file is created by reason of discontinuation of method. Event file records of an events of some duration, or an episode or segment of use or non-use. The terms “event”, “episode”, and “segment” used interchangeably. An episode is defined as uninterrupted period of specific contraceptive method use by women 5 year prior to survey. For instance, if a woman used pill for three months and discontinued, she is said to have contribute an episode. A total 349,236 episodes were formed of which 119,548 were episode of any modern spacing method. The event file can be used to understand contraceptive use dynamics, and particularly contraceptive discontinuation rates, failure rates and switching rates using multiple decrement lifetable analysis. District is the unit of analysis. A district level file has created, which has used the discontinuation rate and other covariates for 640 district of India.

## Method

### Outcome variable

Discontinuation of any modern spacing method in districts of India is the dependent variable in the analysis. The modern spacing method includes pill, IUD, injectable, diaphragm, condom, LAM (Lactation amenorrhea method), foam and jelly and other modern method has included. A 12-month discontinuation rate has calculated for each 640 district of India. Besides, discontinuation of any method is computed for robustness analysis and comparison purpose.

### Independent variables

The independent variables used in the analysis are; mean years of schooling, parity 2+, use of female sterilization, women occupation, unmet need and Method Information Index (MII). The MII has been created by compiling three questions asked to women while they get informed before using of modern method of contraception i.e. “side effect of the method”, “how to manage side effect”, “told about other method” [1, 23]. The index has calculated by assigning (yes = 1, otherwise = 0) for all the above three questions. Summing to all create a variable ranges 0 to 3. The value 3 indicates the user has informed for all the three questions and 0 indicates do not informed about method and 1, 2 indicates partial information.

### Statistical analysis

Descriptive statistics, multiple decrement lifetable, univariate and bivariate analysis of Moran’s I statistics and LISA map has been estimated to assess the spatial autocorrelation and multivariate spatial regression model has used to assess the spatial effect. The 12-month discontinuation rate for any modern spacing method has been calculated by multiple decrement life table. It provides net discontinuation rate among those using the information of episode of contraceptive started began (3–62) months preceding the survey. The episode of the contraceptive can be discontinued due to various reasons and the reason of the discontinuation is considered as competing risk. Twelve-month discontinuation rate can be defined as the cumulative proportion of episode is discontinued for any reason by the twelve months of use. Mathematically cumulative probability of 12-month discontinuation rate is given as in Eq. 1 below.

1 $${\text{Q}}_{{{\text{i}},{\text{ j}}}} \, = \,{\text{1}} - {\text{ }}\prod _{{{\text{i}} = {\text{1}}:{\text{12}}}} \left( {{\text{1}} - {\text{q}}_{{{\text{i}},{\text{ j}}}} } \right)$$

where i denotes month of the year and j denotes reason of the discontinuation.

To explore the pattern of spatial cluster and correlate, bivariate local indicator of spatial association (LISA) map and scatter plot has been used. LISA map indicate the association between district value with lagged district value. The Moran’s I value reflects spatial autocorrelation and varies between -1 to 1. The Moran’s I value close to −1 indicate perfect clustering of dissimilar value (dispersion) while Moran’s value +1 indicate perfect clustering of similar value while value 0 indicates no autocorrelation (perfect randomness). LISA scatter plot was decomposed into four quadrants (Q1, Q2, Q3 & Q4) of association such as Q1(high–high), Q2(low–high), Q3(low–low) & Q4(high–low). Among four quadrant of association Q1 & Q2 have positive spatial association i.e. similar discontinuous rate of districts values positively associated with neighbour districts. Moran’s I statistics equation is given as

$${\text{I}} = \sum {\text{i}}\left( {{\text{zi}} \times \sum {\text{jwijzj}}} \right)/\sum {\text{iz}}^{2}$$

To understand the associated significant factor of contraceptive discontinuation rate, the OLS model and impact analysis has used. To measure the neighbourhood and spill over effect, impact analysis has done whereas direct effects suggest effect from its neighbouring area and indirect effect would suggest spatial spill over effect as well. Total effects is the sum of direct and indirect effect. The impact analysis of spatial autoregressive (SAR) is the robust model as simple OLS gives biased estimate [24–27]. The equation is expressed as follows,

2 $${\text{Y}}_{{\text{i}}} \, = \,\beta {\text{X}}_{{\text{j}}} + \rho {\text{W}}_{{{\text{ij}}}} {\text{Y}}_{{\text{j}}} + {\text{ }}\varepsilon _{{\text{i}}}$$

where ‘Y_i_’ denotes the DMSM and ‘X_j_’ is the set of explanatory variables i.e. mean year schooling, MII, parity 2+, unmet need, female sterilization, method attribute and failure opposition, desire for children, visit health facility, schedule caste (SC)/schedule tribe (ST), urban and occupation. ‘ρ’ is the spatial autoregressive coefficient, ‘W_ij_’ denotes the spatial weights proximity between districts i and j, ‘y_j_’ is the DMSM ‘β’ denotes the regression coefficients and ɛ_i_ is the residual. All the analysis has been performed by using ArcGIS 10.7.1, STATA 15.1 and GeoDa 1.12.1.

## Results

Table 1 presents the mean, coefficient of variation, minimum and maximum value of the variables used in the study. The distributions of the variables suggested wide variation in each of the dependent and independent variables. The mean value for the episode of any method (546) was remarkably higher than the episode of modern spacing method (186). The wide variation was observed for the episode of modern spacing (0.89) than that of any method (0.55). The coefficient of variation for MII was 0.78 followed by percentage of urban population (0.76) and percentage of women with schedule caste (SC) or schedule tribe (ST) (0.63). The least variation was observed for the percentage of women desire for additional children (0.24) followed by DMSM (0.33).

**Table 1:** Descriptive statistics of selected variables in district of India, 2015–16

Variable (district level/percentage)	Mean	Coefficient of variation	Min.	Max.
Episode of modern spacing method	186	0.89	4	1160
Episode of any method	546	0.55	48	2553
Discontinuation of any modern spacing method (%)	47	0.33	8	100
Parity 2+ (%)	24	0.56	0	61
% Women working	24	0.43	3	58
%Women with unmet need	14	0.46	2	35
Mean years schooling (MYS)	1.4	0.35	0	3
Method information index (MII)	22	0.78	0	100
%Using female sterilization	32	0.53	1	76
Method attribute and failure opposition (%)	36	0.39	0	100
%Women desire for additional child	18	0.24	7	40
Visit health facility by any health issue (%)	23	0.55	2	72
%Scheduled caste/schedule tribe	36	0.63	1	100
%Urban	28	0.76	0	100

### Discontinuation pattern of modern spacing method

**Fig. 1:**
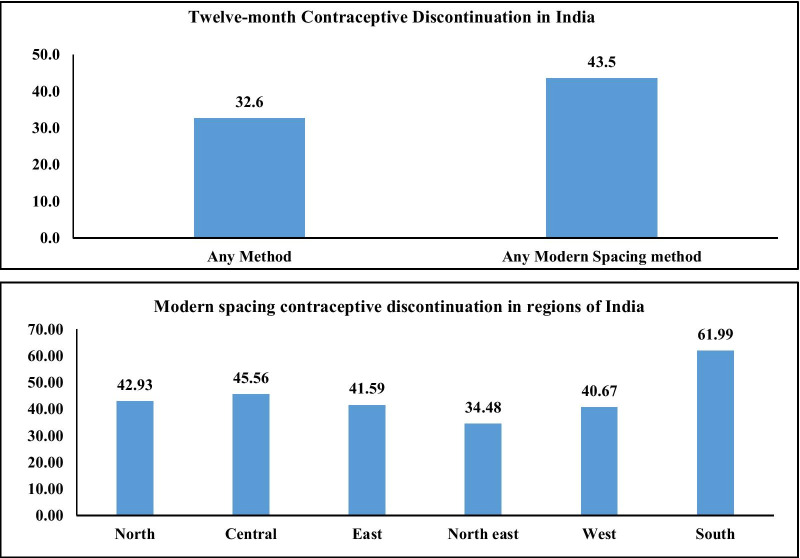
Twelve-month discontinuation rate of modern spacing method and any method for India and regions, 2015–16

Figure 1 presents the DMSM in India and its regions. The discontinuation rate was 32.6% of any method and that for modern spacing method was 43.5%. The discontinuation rate was highest in southern region (61.99%) followed by central (45.56%), North (42.93%), East (41.59%) and North-East (34.48%).

Table 2 presents the 12-month discontinuation rate estimated by reasons of discontinuous in India. Discontinuation rate are presented by any method and any modern spacing method. Desire to become pregnant is the leading cause of discontinuation for any method and any modern spacing method (12.43%), followed by other fertility related reasons and methods relates reason’s (4.40%). Method failure, side effect of method and method related reason together accounts 12% of contraceptive discontinuation in India.

**Table 2:** Twelve-month discontinuation rate (%) by method & reason in India, 2015–16

Reason	Discontinuation rate by any method^a^	Discontinuation rate by any modern spacing method^b^
Method failure	2.38	2.52
Desire to become pregnant	9.33	12.43
Side effects/health concerns	2.62	4.99
Wanted more effective method	0.17	0.27
Other fertility related reasons	5.22	6.34
Other method related	3.17	4.40
Other/DK	9.70	12.57

Figures are based on life table calculations using information on episodes of contraceptive use that began 3–62 months preceding the survey

^a^Discontinuation rate by any method includes male & female sterilizations. Total no. of episode is 349,236. ^b^Modern spacing method includes Pill, Intrauterine device (IUD), Injectable, Diaphragm, Condom, Lactation amenorrhea method (LAM), Foam and Jelly, Other modern method. Total no. of episode is 119,548

Table 3 presents regional variation (group of states) of discontinuation in modern spacing method in India. That southern region has high discontinuation pattern than all other region. Among all the reason of discontinuation ‘desire to become pregnant was highest than ‘Other/DK’, ‘Other fertility related reason’, ‘Side effects/health concern’, ‘Other method related reason’ and lowest reason is wanted more effective method’. Discontinuation due to ‘Desire to become pregnant’ is the highest in South region next to west region and lowest in North-east region.

**Table 3:** Twelve-month discontinuation rate (%) for any modern spacing method in reason of discontinuation given by region of India, 2015–16

Reason	North	Central	East	North East	West	South
Method failure	1.97	3.58	2.47	0.56	3.71	0.48
Desire to become pregnant	12.47	12.81	9.70	7.71	15.55	20.31
Side effects/health concerns	4.88	3.58	6.45	4.34	4.55	5.26
Wanted more effective method	0.32	0.48	0.12	0.19	0.02	0.67
Other fertility related reasons	6.70	7.70	6.24	3.00	3.12	11.3
Other method related	3.93	5.73	5.30	2.51	2.92	2.89
Other/DK	12.65	11.68	11.3	16.16	10.79	21.08
Total no. of episode^a^	38,031	26,997	20,097	22,500	7396	4527

^a^Total number of episode is divided by region of India (Additional file 1: Appendix S4) & it is only for modern spacing contraceptive method

### Spatial heterogeneity of discontinuation of modern spacing method (DMSM) & prevalence of modern spacing method

**Fig. 2:**
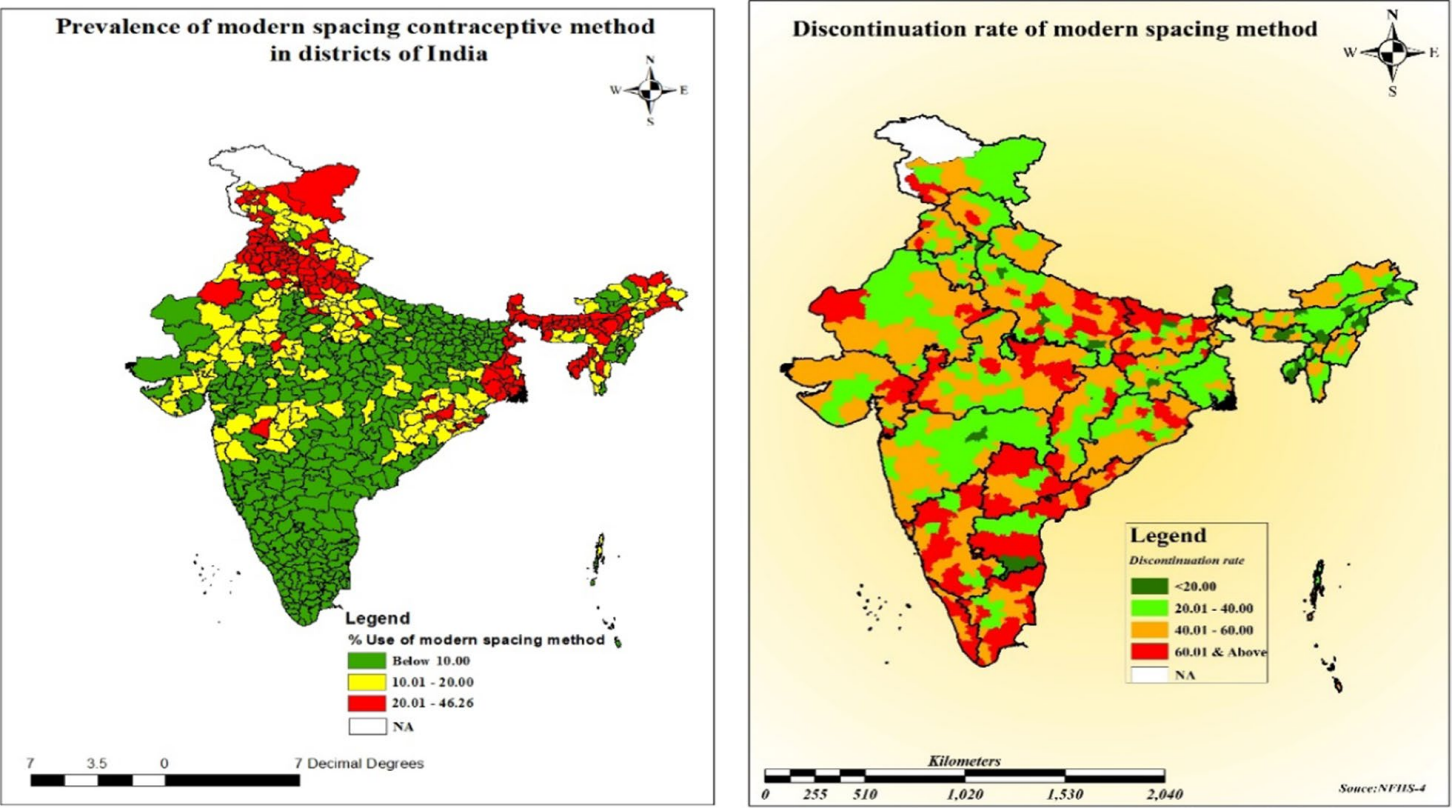
Prevalence of modern spacing method and discontinuation rate of any modern spacing method in districts of India, 2015–2016. Source: Authors are generated from Arc GIS 10.7.1

Figure 2 presents spatial pattern of modern spacing method use and discontinuation of any modern spacing method in 640 district of India (Additional file 1: Appendix S1). The use of any modern spacing method was below 10% in 21 districts (low) 10%–20% in 173 districts (Medium), 20%–46% in 139 high categories. The use of modern spacing methods varies to a large extent across districts of India. DMSM comparatively very high than any other method. The DMSM clustered in southern region, parts of Madhya Pradesh, Uttar Pradesh, Bihar and Odisha. Very low pattern of clustering is observed in North eastern region. Clustering of discontinuation is high in central and southern region. A total of 21 district falls under low category, 211 districts in Middle, 278 districts in high and 130 districts under very high category of contraceptive discontinuation.

### Spatial correlates of discontinuation of modern spacing method

**Fig. 3:**
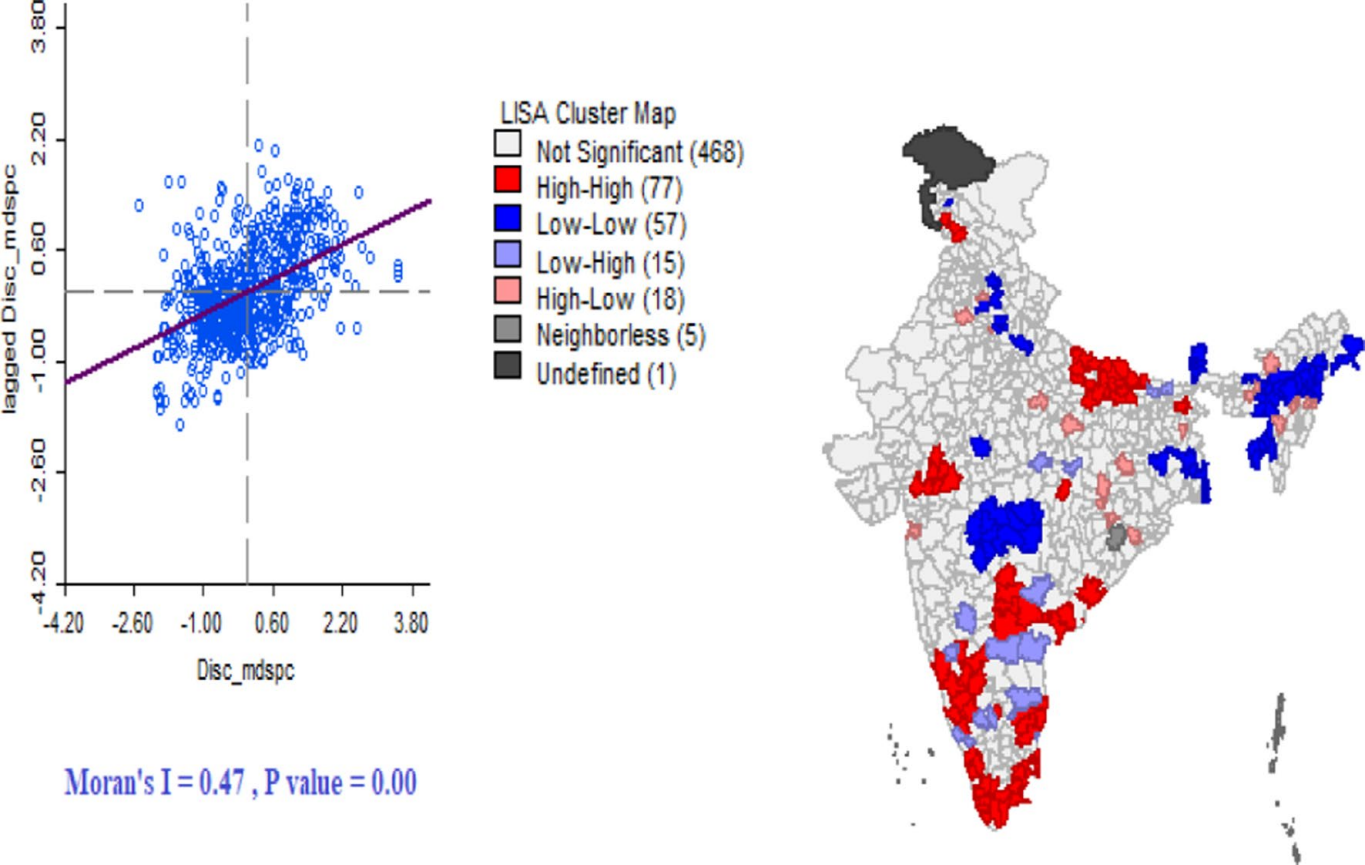
Univariate LISA cluster map of discontinuation rate of modern spacing contraceptive method, 2015–16. Source: Authors are generated from GeoDa version 1.12.1

**Fig. 4:**
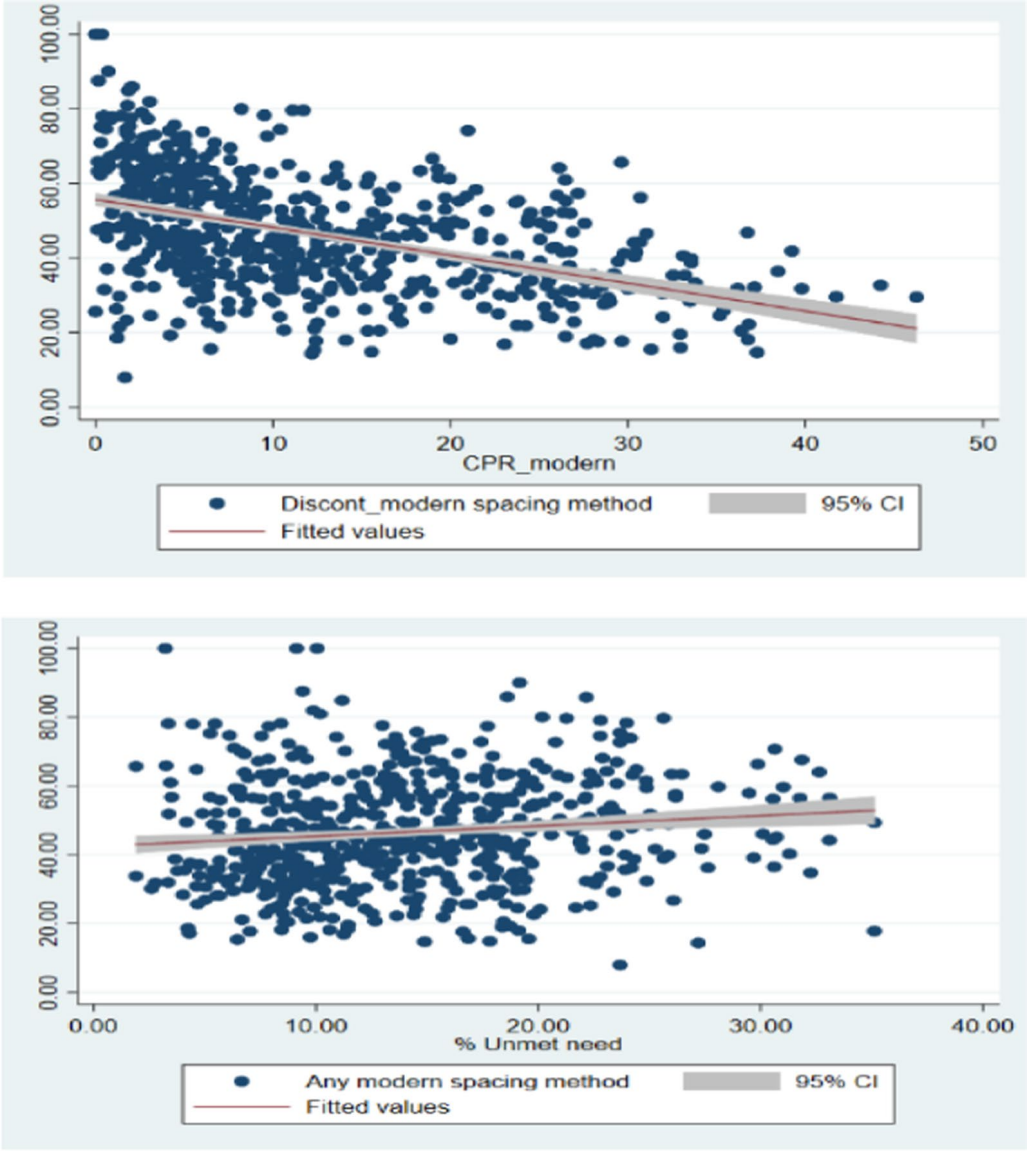
Visualization of scatter plot between discontinuation rate of any modern spacing method between (**a**) prevalence of modern spacing method (**b**) unmet need. Source: Authors are generated graphs using STATA 15.1

**Fig. 5:**
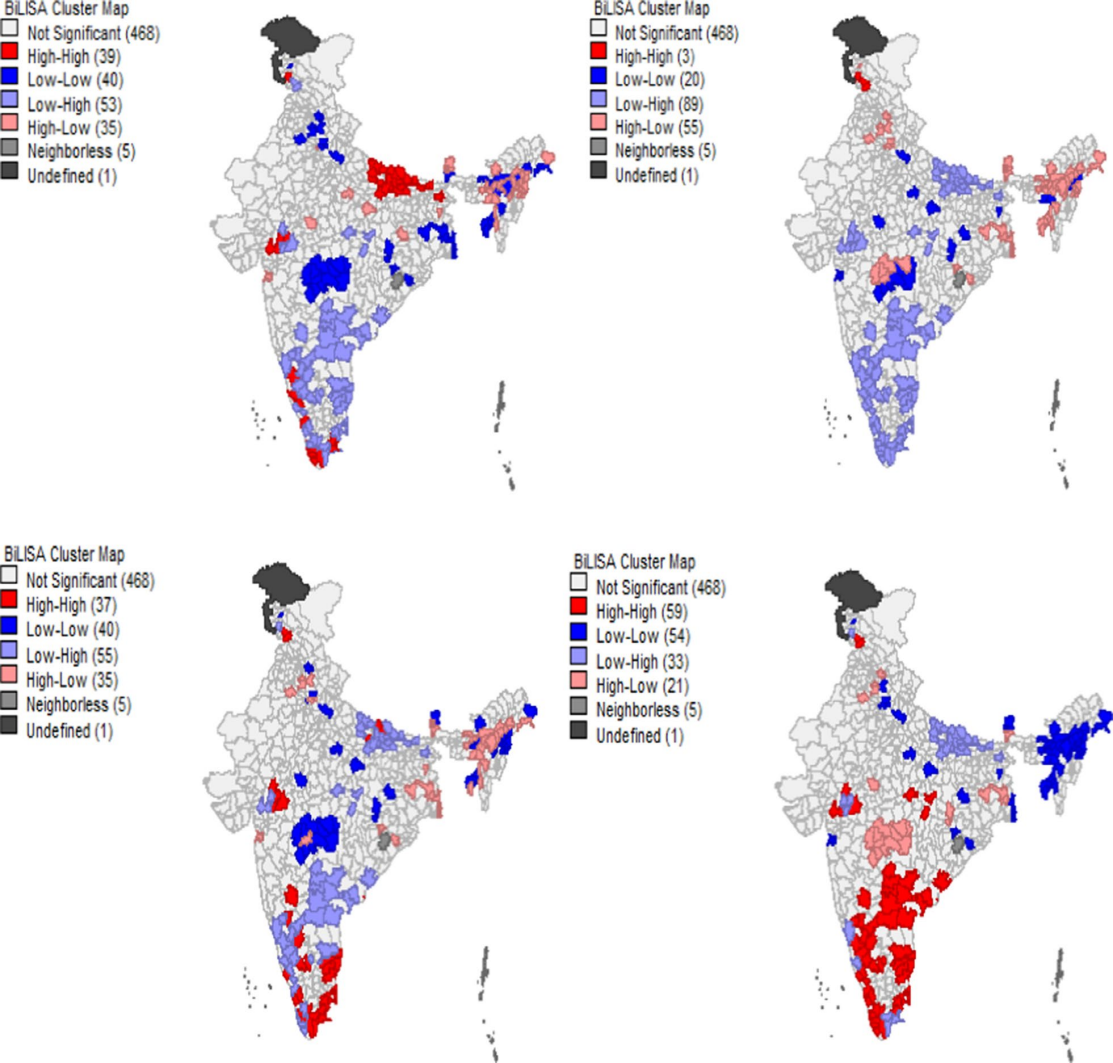
Bivariate LISA cluster map of India representing geographic cluster (hot spot & cold spot) of discontinuation rate of any modern spacing method between (**a**) MII (**b**) prevalence of modern spacing method (**c**) unmet need (**d**) female sterilization, 2015–16. Source: Authors generated these map using GeoDa Version 1.12.1

Figure 3 provides the univariate’s LISA cluster map of discontinuation in any modern spacing method and Moran’s I statistics in districts of India. The Moran’s I value 0.47 suggests significantly positive spatial association in discontinues of modern spacing method. From the LISA cluster map, we found that 77 districts have hotspot that signifies these districts had high discontinuation with their neighbouring districts. These districts have largely from the southern states of India; Karnataka, Kerala, Andhra Pradesh, Tamil Nadu and Uttar Pradesh. Figure 4 provides scatter plot of prevalence of modern spacing method, unmet need with DMSM in the districts of India. Districts with low use of modern contraception tend to have higher discontinuation than districts with low use of modern method. Similarly, the discontinuation of any modern spacing method does not show any pattern with unmet need for spacing. Figure 5 provides Bivariate LISA cluster map of prevalence of modern spacing method, MII, unmet need, female sterilization with DMSM to understand the spatial correlates in districts of India. Figure 5a–d provides the spatial correlation of MII, prevalence of modern spacing method, unmet need and female sterilization with DMSM. In case of modern spacing method, we found only 3 districts (Kathua, Udhamp, Reasi) have hot spot, 20 districts have cold spot and remaining districts have spatial outliers. For MII in 37 districts had hot spot and 40 districts had cold spots; these districts are from the states of Tamil Nadu, Karnataka and Kerala. In case of unmet need 39 districts have hot spot and 40 districts cold spot belonging from Uttar Pradesh and Kerala. For female sterilization 59 districts have hot spot and 54 districts have cold spots these are from Andhra Pradesh, Karnataka, Tamil Nadu and Kerala.

### Global spatial regression model

Additional file 1: Appendix S3 presents results of OLS regression model of discontinuous rate of any modern spacing method with associated covariates. The spatial OLS result suggests significant of spatial autocorrelation (Moran’s I) hence spatial regression model used. From the diagnostic test of spatial autocorrelation, it has confirmed that residual of spatial autocorrelation (Moran’s I) of any modern spacing method is statistically significant (Moran’s I = 0.47, P-value = 0.00).

### Impact analysis

Table 4 presents the results of direct effects, indirect effect and total effect of DMSM with associated covariates. The direct effect presents the marginal effect of the change in the independent variable of one percent on the dependent variable of the same unit. The indirect effect is the marginal effect of the change in the independent variable in one percent on the dependent variable value of all neighboring units. Change in DMSM had the largest direct effect upon unmet need, meaning that higher the DMSM led to higher prevalence of unmet need in whole within the country. For indirect effect/spill over effect of unmet need had not significant to DMSM. The total effects indicate that an increase in unmet need with higher the DMSM value. Female sterilization had positive direct effect on DMSM significantly and total effect of DMSM increases when increase the prevalence of female sterilization but had not significant in indirect effect. There was no significant coefficient in indirect effect meaning that no spill over effects of any of the covariates affecting the DMSM.

**Table 4:** Result of direct, indirect and total impact of Spatial Autoregressive model (SAR) for any modern spacing method in India, 2015–16

	Variables	dy/dx	P-value	95% CI	Significant
Direct	Mean year of Schooling	1.38	0.42	− 2.00	4.75
	MII	− 0.02	0.55	− 0.10	0.05
	Parity2+	− 0.33	0.22	− 0.85	0.20
	Unmet need	0.84	0.00	0.55	1.14***
	Female Sterilization	0.54	0.01	0.14	0.95***
	Method attribute and failure opposition	0.04	0.27	− 0.03	0.12
	Desire of children	0.26	0.10	− 0.05	0.57*
	Visit health facility	− 0.03	0.59	− 0.15	0.09
	Schedule caste/Schedule tribe	− 0.09	0.01	− 0.15	− 0.03***
	Urban	− 0.07	0.02	− 0.14	− 0.01**
	Occupation	0.09	0.18	− 0.04	0.22
Indirect	Mean year of Schooling	− 0.03	0.65	− 0.15	0.09
	MII	0.00	0.69	0.00	0.00
	Parity2+	0.01	0.62	− 0.02	0.03
	Unmet need	− 0.02	0.58	− 0.08	0.04
	Female Sterilization	− 0.01	0.59	− 0.05	0.03
	Method attribute and failure opposition	0.00	0.62	0.00	0.00
	Desire of children	− 0.01	0.61	− 0.03	0.01
	Visit health facility	0.00	0.71	0.00	0.00
	Schedule caste/Schedule tribe	0.00	0.59	0.00	0.01
	Urban	0.00	0.60	0.00	0.01
	Occupation	0.00	0.62	− 0.01	0.01
Total	Mean year of Schooling	1.35	0.43	− 1.96	4.66
	MII	− 0.02	0.55	− 0.10	0.05
	Parity2+	− 0.32	0.22	− 0.84	0.19
	Unmet need	0.82	0.00	0.53	1.12***
	Female Sterilization	0.53	0.01	0.13	0.94***
	Method attribute and failure opposition	0.04	0.28	− 0.03	0.12
	Desire of children	0.26	0.10	− 0.05	0.56*
	Visit health facility	− 0.03	0.59	− 0.15	0.09
	Schedule caste/schedule tribe	− 0.09	0.01	− 0.15	− 0.03***
	Urban	− 0.07	0.02	− 0.13	− 0.01**
	Occupation	0.09	0.18	− 0.04	0.22

^***^p < 0.01, **p < 0.05, *p < 0.1 (indicates statistically significant)

## Discussion

India is the first country in the world to official launch the family planning programme in 1952 with objective to reduce fertility level and achieve population stabilization. Over the years, the family planning programme underwent several changes. Family planning programme is now has been integrated in the broader domain of reproductive and child health programme. The focus of India’s family planning programme has long been on increasing female sterilization. The over emphasis of female sterilization in official family planning programme led to low use of spacing method. Since ICPD, though the Government of India and various state Government emphasize the use of spacing method, still it remained low across the country. Moreover, the discontinuation of spacing method among small users remained high. Thus, contraceptive discontinuation is the major issue of the family planning programme to achieve the goal of family planning in India. Districts in India are the key administrative unit and varies enormously in fertility, contraceptive use and level of development. In this context, this is first ever study that examine the spatial pattern of contraceptive discontinuation in districts of India. This paper has used the calendar data and make a systematic attempt for understanding the spatial heterogeneity and correlates of contraception discontinuation in districts of India. The followings are the salient findings of this study.

First, our findings suggest that districts the low use and high discontinuation of spacing method is spread in districts of poorer and high developed states of India. Though districts in southern region are demographically advanced with higher use of permanent method, the DMSM is also high. The districts of central region such as Uttar Pradesh, Madhya Pradesh and Bihar have high discontinuation rate due to desire for additional child, lack of accessibility and affordability of spacing method and low knowledge of method. The low use and high discontinuation rates are also from the districts of states of Andhra Pradesh, Karnataka, Kerala and Tamil Nadu. Desire for child is the major reason of discontinue followed by fertility related issue and side effect of method were becoming the major issues. The opposition to use of family planning method on religious ground and husband opposition is also one of the reasons of discontinuation [28]. Second, the LISA cluster map identified districts with hot spot, cold spot regions. The hot spots (high-high) regions are those cluster districts which are highly associated with their neighbour districts and cold spots (low-low) are those cluster districts which are less associated with their neighbourhood. Third, our finding suggests that the MII is a significant predictor of discontinuation. Due to lack of comprehensive knowledge the use of modern spacing method and discontinuation is high. So the quality care for family planning services should be improved to expedite the family planning programme in the long way. Despite the major concern of discontinuation there are some natural fertility related reason of discontinuation are infrequent sex, menopause, hysterectomy, infecund and postpartum or breast feeding are also contributing to discontinue. Fourth, our findings shows high discontinuation of injectable, followed by condom, pill and IUD. The method specific discontinuation rate demands for improving the quality of services.

Our findings suggest that programmatic attention on increasing use of modern spacing method and reducing discontinuation should be given to districts with low prevalence of modern spacing method and high discontinuation, so that it led to reduce the health issue and unintended pregnancy and also government encouraging to spacing the child birth not limiting. The discontinuation for injectable (51%) is as high as followed by condom (47%), pill (42%) and IUD (26%) captured through national survey and it conceal the large variation across the districts of India [17]. Often women complain side effect of IUD as reason for discontinuation. In case of pill and other modern spacing method, the continuity in use need to be promoted. The method related barriers should be addressed. Increasing use and high discontinuation of traditional method in some districts of India is a major concern. Motivating couples to switch from traditional to modern spacing method is possible and yield increase use and retention of spacing method. The follow up counselling and health worker visit to households may be promoted to increase the awareness for spacing approach method. It may be mentioned that with implementation of National Health Mission (NHM), the contact of ASHA and mothers has increased in the country. It is suggested that the ASHA worker may be trained for counselling and follow up of spacing method. Our result suggests that unmet need have strong positive association with discontinuation of modern spacing. Thus women with unmet need may be motivated for use of spacing method. Similarly, before young mothers are sterilised, they may be informed about various alternative spacing method. The misconception on spacing method may be reduced through mass-media and home visit of health worker. It is established that parity 2+, desire of children is the important predictor of discontinuation as it has supported the previous studies [19, 28, 29]. Policy implication could have emphasized these two predicators, so that it can reduce the discontinuation of contraceptive methods.

## Conclusion

The current family planning programme should focus on increasing use of modern spacing method than limiting approach and motivating traditional method user to use modern spacing method. The high discontinuation pattern in certain districts need more intervention. The districts identified as hot spot and cold spot should be prioritise in programme and policy for family planning. More attention for health counselling for client prospective, health worker outreach to user and better quality care services will stimulate non-user of contraception to use of modern spacing method. This will enable to achieve many of the health related SDGs.

## Strength and limitations

The main strength of the study is estimation of discontinuation of modern spacing contraceptive method from calendar data of NFHS-4, 2015–16. Univariate and bivariate spatial analysis portray the spatial correlation of neighbouring districts which will be new finding study ever. The study could not include some independent variable due to insufficient sample size and data constraint. These variables include contraceptive intention for spacing or limiting, family planning services, age specific analysis and supply side constraints. This study focus only 12-month discontinuation rate as it further studies can be 24 months and 36 months.

## Supplementary Information

**Additional file 1: Appendix S1.** Sample distribution of number of women, number of episode, prevalence and discontinuation rate of any method and modern spacing method of contraception of 640 districts of India, 2015–16. **Appendix S2.** Result of Bivariate relationship between high modern spacing contraceptive prevalence rate with low discontinuation of modern spacing method and low modern spacing contraceptive prevalence rate with high discontinuation of modern spacing method from LISA cluster map, 2015–16. **Appendix S3.** Estimated result of spatial weighted OLS regression model for any modern spacing contraceptive method, 2015–16. **Appendix S4.** Classification of states by region in India, NFHS-4(2015–16).

## Data Availability

The unit level data is available from the Demographic Health Survey (DHS) data repository through https://dhsprogram.com/data/available-datasets.cfm and could be accessed upon a data request subject to non-profit and academic interest only.

## Abbreviations

NFHS: National family health survey
LISA: Local indicator of spatial autocorrelation
MDG: Millennium development Goal
SDG: Sustainable development Goal
MII: Method information index
SEM: Spatial error model
OLS: Ordinary least square
SAR: Spatial autoregressive model
DMSM: discontinuation of modern spacing method
IUD: Intrauterine device
USAID: United State Agency for International Development
