# Supplementary material for: Spatial heterogeneity in discontinuation of modern spacing method in districts of India
Source: Reprod Health. 2021 Jun 30;18:137. doi: 10.1186/s12978-021-01185-w (PMC8244153; doi:10.1186/s12978-021-01185-w)
Supplement: Supplementary file 1 — Additional file 1: Appendix S1. Sample distribution of number of women, number of episode, prevalence and discontinuation rate of any method and modern spacing method of contraception of 640 districts of India, 2015–16. Appendix S2. Result of Bivariate relationship between high modern spacing contraceptive prevalence rate with low discontinuation of modern spacing method and low modern spacing contraceptive prevalence rate with high discontinuation of modern spacing method from LISA cluster map, 2015–16. Appendix S3. Estimated result of spatial weighted OLS regression model for any modern spacing contraceptive method, 2015–16. Appendix S4. Classification of states by region in India, NFHS-4(2015–16). [file 12978_2021_1185_MOESM1_ESM.docx]

**Appendix S1: Sample distribution of number of women, number of episode, prevalence and discontinuation rate of any method and modern spacing method of contraception of 640 districts of India, 2015-16**

|  |  | **Any Method** | | | | **Modern Spacing Method** | | | |
| --- | --- | --- | --- | --- | --- | --- | --- | --- | --- |
| **Number of Districts** | **Name of Districts** | **Number of women** | **Number of episode** | **Prevalence of any method** | **Discontinuation rate of any method** | **Number of women** | **Number of episode** | **Prevalence of modern spacing method** | **Discontinuation rate of modern spacing method** |
|  | **Jammu & Kashmir** |  |  |  |  |  |  |  |  |
| 1 | Kupwara | 1098 | 648 | 50.92 | 44.88 | 1,098 | 288 | 16.3 | 55.47 |
| 2 | Badgam | 1253 | 775 | 69.05 | 29.26 | 1,253 | 412 | 28.08 | 30.45 |
| 3 | Leh | 807 | 571 | 70.25 | 27.97 | 807 | 429 | 46.26 | 29.50 |
| 4 | Kargil | 1032 | 645 | 62.86 | 40.27 | 1,032 | 447 | 39.24 | 41.88 |
| 5 | Punch | 1387 | 1018 | 48.6 | 67.37 | 1,387 | 669 | 20.98 | 74.20 |
| 6 | Rajouri | 1128 | 458 | 28.01 | 74.49 | 1,128 | 289 | 11.71 | 79.61 |
| 7 | Kathua | 1081 | 870 | 74.11 | 51.79 | 1,081 | 395 | 26.5 | 55.38 |
| 8 | Baramula | 1062 | 470 | 58.55 | 28.11 | 1,062 | 217 | 21.11 | 35.83 |
| 9 | Bandipore | 1201 | 598 | 53.96 | 36.87 | 1,201 | 241 | 15.24 | 46.21 |
| 10 | Srinagar | 982 | 595 | 68.44 | 31.72 | 982 | 300 | 25.8 | 30.37 |
| 11 | Ganderbal | 1151 | 820 | 60.14 | 41.77 | 1,151 | 381 | 21.56 | 38.90 |
| 12 | Pulwama | 1078 | 609 | 68.97 | 21.59 | 1,078 | 183 | 15.86 | 33.36 |
| 13 | Shupiyan | 1184 | 618 | 66.28 | 17.08 | 1,184 | 189 | 16.92 | 24.27 |
| 14 | Anantnag | 1162 | 571 | 53.95 | 29.17 | 1,162 | 293 | 22.28 | 38.05 |
| 15 | Kulgam | 1187 | 644 | 60.41 | 30.32 | 1,187 | 324 | 25.38 | 36.18 |
| 16 | Doda | 933 | 310 | 30.44 | 61.87 | 933 | 141 | 9.68 | 72.68 |
| 17 | Ramban | 945 | 462 | 41.5 | 53.56 | 945 | 233 | 15.41 | 61.65 |
| 18 | Kishtwar | 929 | 466 | 55.39 | 29.58 | 929 | 177 | 16.42 | 45.56 |
| 19 | Udhampur | 1098 | 922 | 70.22 | 50.05 | 1,098 | 399 | 22.04 | 52.68 |
| 20 | Reasi | 1210 | 1008 | 53.33 | 65.37 | 1,210 | 483 | 19.29 | 63.83 |
| 21 | Jammu | 944 | 804 | 63.79 | 64.44 | 944 | 437 | 26.13 | 64.16 |
| 22 | Samba | 948 | 352 | 31.23 | 56.17 | 948 | 219 | 13.58 | 64.62 |
|  | **Himachal Pradesh** |  |  |  |  |  |  |  |  |
| 23 | Chamba | 925 | 412 | 51.61 | 33.66 | 925 | 127 | 11.45 | 50.35 |
| 24 | Kangra | 850 | 479 | 47.92 | 47.96 | 850 | 211 | 16.14 | 55.24 |
| 25 | Lahul and spiti | 592 | 344 | 62 | 25.56 | 592 | 125 | 14.53 | 37.68 |
| 26 | Kullu | 889 | 556 | 63.43 | 34.44 | 889 | 214 | 16.79 | 44.76 |
| 27 | Mandi | 856 | 607 | 65.55 | 43.48 | 856 | 164 | 9.89 | 62.79 |
| 28 | Hamirpur | 830 | 390 | 44.03 | 38.3 | 830 | 158 | 11.64 | 49.10 |
| 29 | Una | 881 | 307 | 33.94 | 37.23 | 881 | 189 | 16.72 | 45.80 |
| 30 | Bilaspur | 835 | 427 | 52.05 | 34.86 | 835 | 135 | 9.57 | 57.09 |
| 31 | Solan | 877 | 645 | 72.38 | 32.59 | 877 | 228 | 16.49 | 43.06 |
| 32 | Sirmaur | 957 | 497 | 60.47 | 29.1 | 957 | 172 | 16.33 | 41.04 |
| 33 | Shimla | 770 | 558 | 73.25 | 26.1 | 770 | 227 | 24.04 | 31.71 |
| 34 | Kinnaur | 667 | 473 | 68.58 | 27.55 | 667 | 202 | 21.01 | 30.28 |
|  | **Punjab** |  |  |  |  |  |  |  |  |
| 35 | Gurdaspur | 941 | 936 | 74.45 | 63.5 | 941 | 418 | 26.02 | 56.80 |
| 36 | Kapurthala | 960 | 931 | 70.02 | 58.73 | 960 | 442 | 23.83 | 55.34 |
| 37 | Jalandhar | 986 | 906 | 70.56 | 34.78 | 986 | 458 | 28.55 | 32.48 |
| 38 | Hoshiarpur | 925 | 851 | 70.32 | 49.05 | 925 | 419 | 25.06 | 45.01 |
| 39 | Sangrur | 881 | 667 | 64.65 | 38.39 | 881 | 328 | 25.74 | 42.69 |
| 40 | Fatehgarh sahib | 864 | 867 | 73.84 | 58.81 | 864 | 370 | 30.71 | 44.38 |
| 41 | Ludhiana | 918 | 780 | 72.75 | 31.79 | 918 | 352 | 26.88 | 26.98 |
| 42 | Moga | 1003 | 1000 | 76.64 | 45.14 | 1,003 | 498 | 29.78 | 41.14 |
| 43 | Firozpur | 1022 | 1049 | 80.85 | 49.41 | 1,022 | 520 | 25.08 | 49.50 |
| 44 | Muktsar | 975 | 1151 | 84.81 | 67.29 | 975 | 541 | 29.63 | 65.67 |
| 45 | Faridkot | 1026 | 1222 | 81.48 | 51.72 | 1,026 | 588 | 25.62 | 48.53 |
| 46 | Bathinda | 1033 | 1110 | 82.61 | 43.19 | 1,033 | 556 | 33.22 | 35.35 |
| 47 | Mansa | 1013 | 1040 | 78.33 | 50.54 | 1,013 | 516 | 26.75 | 52.00 |
| 48 | Patiala | 1042 | 1028 | 79.13 | 41.08 | 1,042 | 519 | 33.57 | 38.76 |
| 49 | Amritsar | 1051 | 987 | 80.96 | 31.27 | 1,051 | 498 | 33.5 | 28.42 |
| 50 | Tarn taran | 1019 | 1141 | 80.84 | 63.55 | 1,019 | 527 | 26.47 | 60.96 |
| 51 | Rupnagar | 925 | 861 | 75 | 43.12 | 925 | 425 | 30.37 | 40.41 |
| 52 | Sahibzada ajit s | 880 | 947 | 75.45 | 55.03 | 880 | 413 | 27.56 | 49.31 |
| 53 | Shahid bhagat si | 1050 | 844 | 65.58 | 47.11 | 1,050 | 448 | 26.89 | 47.00 |
| 54 | Barnala | 970 | 1007 | 78.61 | 54.18 | 970 | 441 | 24.52 | 52.14 |
|  | **Chandigarh** |  |  |  |  |  |  |  |  |
| 55 | Chandigarh | 746 | 619 | 73.99 | 33.12 | 746 | 329 | 36.33 | 29.53 |
|  | **Uttarakhand** |  |  |  |  |  |  |  |  |
| 56 | Uttarkashi | 994 | 655 | 68.54 | 38.73 | 994 | 214 | 13.75 | 46.84 |
| 57 | Chamoli | 849 | 446 | 60.24 | 17.82 | 849 | 112 | 10.27 | 34.50 |
| 58 | Rudraprayag | 905 | 452 | 60.97 | 14.07 | 905 | 122 | 12.1 | 26.70 |
| 59 | Tehri garhwal | 984 | 588 | 64.33 | 33.72 | 984 | 246 | 19.17 | 48.11 |
| 60 | Dehradun | 2032 | 1065 | 59.83 | 26.13 | 2,032 | 570 | 28.89 | 29.33 |
| 61 | Garhwal | 939 | 607 | 67.23 | 40 | 939 | 270 | 20.22 | 49.90 |
| 62 | Pithoragarh | 862 | 583 | 70.13 | 39.26 | 862 | 206 | 15.62 | 52.22 |
| 63 | Bageshwar | 1072 | 566 | 57.88 | 34.64 | 1,072 | 188 | 11.27 | 55.74 |
| 64 | Almora | 931 | 492 | 57.1 | 32.35 | 931 | 197 | 15.54 | 44.54 |
| 65 | Champawat | 996 | 555 | 63.25 | 38.11 | 996 | 189 | 14.02 | 59.54 |
| 66 | Nainital | 2170 | 1048 | 48.47 | 47.94 | 2,170 | 615 | 20.9 | 56.56 |
| 67 | Udham singh naga | 2245 | 947 | 37.98 | 52.65 | 2,245 | 659 | 21.44 | 58.33 |
| 68 | Hardwar | 2321 | 1118 | 41.51 | 47.06 | 2,321 | 789 | 24.4 | 50.72 |
|  | **Haryana** |  |  |  |  |  |  |  |  |
| 69 | Panchkula | 953 | 730 | 79.7 | 28.04 | 953 | 354 | 32.99 | 31.85 |
| 70 | Ambala | 1062 | 886 | 77.6 | 32.32 | 1,062 | 410 | 24.36 | 33.86 |
| 71 | Yamunanagar | 1096 | 642 | 72.95 | 13.84 | 1,096 | 277 | 27.71 | 17.15 |
| 72 | Kurukshetra | 1010 | 731 | 73.99 | 42.1 | 1,010 | 352 | 26.34 | 52.26 |
| 73 | Kaithal | 1007 | 759 | 76 | 26.85 | 1,007 | 394 | 33.81 | 33.25 |
| 74 | Karnal | 990 | 721 | 75.41 | 25.03 | 990 | 347 | 27.57 | 30.73 |
| 75 | Panipat | 954 | 712 | 78.12 | 34.04 | 954 | 253 | 22.49 | 37.69 |
| 76 | Sonipat | 1016 | 771 | 77.76 | 37.96 | 1,016 | 260 | 19.94 | 48.91 |
| 77 | Jind | 1076 | 844 | 76.63 | 29.78 | 1,076 | 311 | 21.74 | 32.03 |
| 78 | Fatehabad | 1104 | 792 | 76.88 | 22.56 | 1,104 | 311 | 22.78 | 30.23 |
| 79 | Sirsa | 1101 | 830 | 73.52 | 28.48 | 1,101 | 387 | 26.83 | 35.56 |
| 80 | Hisar | 1089 | 775 | 72.58 | 41.3 | 1,089 | 324 | 19.71 | 56.17 |
| 81 | Bhiwani | 1101 | 809 | 73.54 | 32.67 | 1,101 | 303 | 22.88 | 40.19 |
| 82 | Rohtak | 990 | 646 | 69.68 | 24.21 | 990 | 304 | 25.41 | 32.59 |
| 83 | Jhajjar | 1031 | 794 | 74.43 | 44.73 | 1,031 | 276 | 16.81 | 59.05 |
| 84 | Mahendragarh | 1108 | 749 | 70.8 | 33.88 | 1,108 | 255 | 17.55 | 46.11 |
| 85 | Rewari | 1011 | 449 | 47.79 | 23.39 | 1,011 | 150 | 13.19 | 31.15 |
| 86 | Gurgaon | 922 | 496 | 51.14 | 45.9 | 922 | 225 | 18.55 | 54.10 |
| 87 | Mewat | 1059 | 156 | 15.54 | 46.46 | 1,059 | 74 | 5.69 | 59.66 |
| 88 | Faridabad | 966 | 250 | 28.33 | 36.54 | 966 | 144 | 12.51 | 43.63 |
| 89 | Palwal | 1008 | 287 | 31.27 | 25.6 | 1,008 | 102 | 8.64 | 39.76 |
|  | **NCR of Delhi** |  |  |  |  |  |  |  |  |
| 90 | North west | 607 | 312 | 53.79 | 27.4 | 607 | 178 | 25.55 | 33.95 |
| 91 | North | 669 | 305 | 45.98 | 39.07 | 669 | 183 | 20.67 | 49.15 |
| 92 | North east | 847 | 423 | 53.96 | 29.82 | 847 | 309 | 36.19 | 31.83 |
| 93 | East | 615 | 235 | 38.05 | 49.4 | 615 | 164 | 23.66 | 54.87 |
| 94 | New Delhi | 534 | 163 | 42.3 | 13.93 | 534 | 63 | 16.01 | 20.54 |
| 95 | Central | 663 | 257 | 48 | 18.6 | 663 | 153 | 24.24 | 21.85 |
| 96 | West | 690 | 374 | 61.65 | 21.56 | 690 | 223 | 31.97 | 24.19 |
| 97 | South west | 693 | 397 | 65.59 | 24.69 | 693 | 211 | 32.55 | 35.40 |
| 98 | South | 596 | 240 | 48.13 | 26.25 | 596 | 132 | 23.67 | 36.37 |
|  | **Rajasthan** |  |  |  |  |  |  |  |  |
| 99 | Ganganagar | 1225 | 795 | 71.14 | 19.38 | 1,225 | 246 | 17.22 | 22.82 |
| 100 | Hanumangarh | 1122 | 728 | 70.58 | 26.71 | 1,122 | 205 | 13.75 | 39.15 |
| 101 | Bikaner | 2456 | 1723 | 71.44 | 29.72 | 2,456 | 731 | 22.75 | 39.92 |
| 102 | Churu | 1144 | 595 | 52.39 | 36.77 | 1,144 | 155 | 8.91 | 47.65 |
| 103 | Jhunjhunun | 1234 | 723 | 63.65 | 34.79 | 1,234 | 199 | 10.64 | 44.84 |
| 104 | Alwar | 1113 | 626 | 59.75 | 23.37 | 1,113 | 141 | 9.14 | 41.53 |
| 105 | Bharatpur | 1077 | 458 | 44.63 | 31.18 | 1,077 | 122 | 8.95 | 52.20 |
| 106 | Dhaulpur | 1142 | 707 | 53.73 | 49.66 | 1,142 | 155 | 7.59 | 69.50 |
| 107 | Karauli | 1100 | 712 | 56.01 | 47.51 | 1,100 | 150 | 6.71 | 70.88 |
| 108 | Sawai madhopur | 1115 | 540 | 50.37 | 36.9 | 1,115 | 171 | 9.29 | 57.85 |
| 109 | Dausa | 1097 | 548 | 54.81 | 35.08 | 1,097 | 119 | 6.23 | 67.06 |
| 110 | Jaipur | 2334 | 1503 | 66.69 | 35.47 | 2,334 | 583 | 17.98 | 46.88 |
| 111 | Sikar | 1261 | 738 | 59.77 | 33.56 | 1,261 | 283 | 14.25 | 42.69 |
| 112 | Nagaur | 1167 | 570 | 54.7 | 12.71 | 1,167 | 142 | 9.15 | 35.04 |
| 113 | Jodhpur | 2265 | 1278 | 61.21 | 27.04 | 2,265 | 449 | 16.31 | 39.49 |
| 114 | Jaisalmer | 1017 | 602 | 53.46 | 41.73 | 1,017 | 137 | 8.32 | 62.64 |
| 115 | Barmer | 963 | 465 | 46.17 | 25.52 | 963 | 89 | 5.67 | 44.53 |
| 116 | Jalor | 1122 | 591 | 58.99 | 21.4 | 1,122 | 115 | 7.07 | 44.00 |
| 117 | Sirohi | 996 | 459 | 47.54 | 35.94 | 996 | 130 | 10.82 | 38.89 |
| 118 | Pali | 1061 | 661 | 57.06 | 45.23 | 1,061 | 217 | 11.95 | 56.54 |
| 119 | Ajmer | 2335 | 1441 | 68.67 | 20.17 | 2,335 | 472 | 16.64 | 28.91 |
| 120 | Tonk | 1084 | 670 | 65.97 | 30.69 | 1,084 | 164 | 13.96 | 34.51 |
| 121 | Bundi | 985 | 506 | 57.67 | 26.44 | 985 | 136 | 12.43 | 35.11 |
| 122 | Bhilwara | 1011 | 548 | 57.04 | 30.98 | 1,011 | 134 | 10.64 | 41.45 |
| 123 | Rajsamand | 1060 | 675 | 61.22 | 34.66 | 1,060 | 312 | 19.87 | 46.16 |
| 124 | Dungarpur | 1087 | 658 | 64.24 | 24.38 | 1,087 | 214 | 14.94 | 35.32 |
| 125 | Banswara | 1100 | 549 | 54.91 | 27.82 | 1,100 | 124 | 8.38 | 51.45 |
| 126 | Chittaurgarh | 872 | 389 | 47.25 | 20.99 | 872 | 97 | 9.15 | 40.25 |
| 127 | Kota | 2156 | 1498 | 71.28 | 30.8 | 2,156 | 463 | 20.19 | 33.33 |
| 128 | Baran | 1173 | 655 | 65.43 | 22.96 | 1,173 | 165 | 11.4 | 41.56 |
| 129 | Jhalawar | 1038 | 642 | 68.24 | 24.5 | 1,038 | 169 | 12.54 | 42.47 |
| 130 | Udaipur | 990 | 648 | 51.18 | 47.85 | 990 | 175 | 12.47 | 46.06 |
| 131 | Pratapgarh | 1063 | 641 | 63.6 | 20.59 | 1,063 | 157 | 10.16 | 37.71 |
|  | **Uttar Pradesh** |  |  |  |  |  |  |  |  |
| 132 | Saharanpur | 2487 | 1659 | 65.01 | 35.13 | 2,487 | 869 | 29.22 | 35.77 |
| 133 | Muzaffarnagar | 1251 | 783 | 62.47 | 26.91 | 1,251 | 401 | 28.98 | 28.25 |
| 134 | Bijnor | 1265 | 580 | 53 | 33.8 | 1,265 | 324 | 26.7 | 37.95 |
| 135 | Moradabad | 2487 | 1487 | 61.01 | 33.34 | 2,487 | 778 | 27.61 | 33.79 |
| 136 | Rampur | 1312 | 888 | 62.52 | 32.47 | 1,312 | 294 | 21.94 | 26.72 |
| 137 | Jyotiba phule na | 1271 | 668 | 59.35 | 20.49 | 1,271 | 391 | 32.94 | 19.57 |
| 138 | Meerut | 3334 | 2553 | 65.32 | 39.49 | 3,334 | 1160 | 26.34 | 41.68 |
| 139 | Baghpat | 1239 | 829 | 68.23 | 32.56 | 1,239 | 309 | 23.22 | 38.49 |
| 140 | Ghaziabad | 2281 | 1609 | 65.87 | 38.91 | 2,281 | 853 | 33.57 | 39.80 |
| 141 | Gautam buddha na | 2194 | 2013 | 74.58 | 40.91 | 2,194 | 814 | 26.75 | 41.80 |
| 142 | Bulandshahr | 1259 | 671 | 57.8 | 27.05 | 1,259 | 327 | 24.43 | 30.23 |
| 143 | Aligarh | 2477 | 1788 | 58.74 | 41.75 | 2,477 | 839 | 22.71 | 40.30 |
| 144 | Mahamaya nagar | 1135 | 843 | 60.09 | 38.66 | 1,135 | 295 | 16.5 | 45.48 |
| 145 | Mathura | 1220 | 642 | 57.09 | 29.49 | 1,220 | 230 | 16.6 | 42.02 |
| 146 | Agra | 2448 | 1573 | 60.84 | 41.6 | 2,448 | 666 | 21.68 | 45.05 |
| 147 | Firozabad | 2506 | 1175 | 47.29 | 39.71 | 2,506 | 483 | 15.24 | 45.50 |
| 148 | Manipuri | 1137 | 452 | 40.46 | 38.92 | 1,137 | 204 | 16.54 | 40.00 |
| 149 | Budaun | 1113 | 770 | 51.61 | 40.8 | 1,113 | 191 | 11.41 | 40.27 |
| 150 | Bareilly | 2506 | 1731 | 64.89 | 37.85 | 2,506 | 772 | 25.33 | 36.33 |
| 151 | Pili hit | 1152 | 727 | 62.93 | 25.28 | 1,152 | 343 | 26.81 | 31.01 |
| 152 | Shahjahanpur | 1064 | 472 | 50.59 | 19.04 | 1,064 | 179 | 17.13 | 26.40 |
| 153 | Kheri | 1046 | 312 | 30.78 | 33.64 | 1,046 | 103 | 7.63 | 51.87 |
| 154 | Sitapur | 985 | 429 | 42.83 | 29.88 | 985 | 143 | 10.05 | 45.52 |
| 155 | Hardoi | 1000 | 369 | 26.35 | 68.91 | 1,000 | 240 | 11.11 | 79.63 |
| 156 | Unnao | 1097 | 376 | 41.78 | 21.78 | 1,097 | 134 | 12.2 | 37.41 |
| 157 | Lucknow | 1862 | 729 | 51.64 | 21.24 | 1,862 | 324 | 21.88 | 26.76 |
| 158 | Rae bareli | 1123 | 228 | 27.75 | 17.71 | 1,123 | 81 | 7.66 | 32.60 |
| 159 | Farrukhabad | 1159 | 638 | 45.8 | 36.06 | 1,159 | 255 | 15.43 | 42.19 |
| 160 | Kannauj | 1134 | 522 | 36.45 | 56.84 | 1,134 | 252 | 13.59 | 62.19 |
| 161 | Etawah | 1145 | 697 | 51.38 | 38.51 | 1,145 | 194 | 10.7 | 44.21 |
| 162 | Auraiya | 971 | 441 | 44.51 | 47.66 | 971 | 152 | 12.34 | 55.46 |
| 163 | Kanpur dehat | 965 | 415 | 47.68 | 27.01 | 965 | 117 | 10.33 | 42.62 |
| 164 | Kanpur nagar | 1901 | 915 | 59.84 | 14.16 | 1,901 | 354 | 23.05 | 16.88 |
| 165 | Jalaun | 894 | 373 | 47.44 | 41.02 | 894 | 101 | 10.47 | 52.87 |
| 166 | Jhansi | 1955 | 1046 | 65.85 | 17.61 | 1,955 | 207 | 10.45 | 31.86 |
| 167 | Lalitpur | 900 | 528 | 68.83 | 13.35 | 900 | 58 | 6.25 | 30.48 |
| 168 | Hamirpur | 871 | 346 | 43 | 38.3 | 871 | 137 | 10.87 | 65.12 |
| 169 | Mahoba | 908 | 523 | 63.95 | 28.42 | 908 | 111 | 10.91 | 36.01 |
| 170 | Banda | 789 | 378 | 54.35 | 17.35 | 789 | 80 | 10.62 | 20.66 |
| 171 | Chitrakoot | 1089 | 399 | 45.82 | 13.63 | 1,089 | 66 | 6.21 | 29.95 |
| 172 | Fatehpur | 923 | 346 | 41.44 | 24.32 | 923 | 96 | 6.96 | 42.06 |
| 173 | Pratapgarh | 1216 | 401 | 38.29 | 44.74 | 1,216 | 95 | 4.41 | 66.89 |
| 174 | Kaushambi | 1026 | 324 | 35.28 | 37.18 | 1,026 | 90 | 6.99 | 60.68 |
| 175 | Allahabad | 1162 | 329 | 37.72 | 19.97 | 1,162 | 84 | 8.41 | 36.71 |
| 176 | Bara banki | 987 | 356 | 37.94 | 29.34 | 987 | 134 | 12.95 | 41.43 |
| 177 | Faizabad | 1202 | 506 | 43.84 | 41.97 | 1,202 | 155 | 10.72 | 49.97 |
| 178 | Ambedkar nagar | 1407 | 510 | 35.56 | 38.37 | 1,407 | 167 | 8.08 | 49.45 |
| 179 | Sultanpur | 1151 | 390 | 32.34 | 63.83 | 1,151 | 184 | 10.41 | 74.49 |
| 180 | Bahraich | 1021 | 130 | 10.71 | 46.89 | 1,021 | 69 | 4.7 | 56.47 |
| 181 | Shrawasti | 1055 | 128 | 8.44 | 49.74 | 1,055 | 61 | 2.61 | 70.68 |
| 182 | Balrampur | 1260 | 48 | 2.67 | 63.54 | 1,260 | 40 | 1.78 | 67.57 |
| 183 | Gonda | 1216 | 184 | 13.78 | 47.75 | 1,216 | 94 | 4.34 | 64.01 |
| 184 | Siddharth nagar | 1236 | 430 | 27.77 | 48.98 | 1,236 | 172 | 9.13 | 57.91 |
| 185 | Basti | 1243 | 284 | 18.33 | 55.27 | 1,243 | 178 | 7.6 | 66.31 |
| 186 | Sant kabir nagar | 1349 | 321 | 21.85 | 49.17 | 1,349 | 111 | 6.19 | 59.75 |
| 187 | Mahrajganj | 1346 | 339 | 27.61 | 33.99 | 1,346 | 114 | 4.95 | 61.03 |
| 188 | Gorakhpur | 1212 | 531 | 44.96 | 41.88 | 1,212 | 157 | 8.8 | 54.23 |
| 189 | Kushinagar | 1360 | 369 | 29.3 | 44.36 | 1,360 | 115 | 5.31 | 68.08 |
| 190 | Deoria | 1289 | 473 | 32.34 | 63.62 | 1,289 | 214 | 9.51 | 78.27 |
| 191 | Azamgarh | 1303 | 447 | 37.14 | 46.24 | 1,303 | 120 | 8.21 | 56.70 |
| 192 | Mau | 1359 | 463 | 34.76 | 48.32 | 1,359 | 106 | 5.32 | 64.90 |
| 193 | Ballia | 1329 | 409 | 32.92 | 38.3 | 1,329 | 91 | 4.17 | 51.83 |
| 194 | Jaunpur | 1318 | 537 | 38.07 | 48.62 | 1,318 | 155 | 6.05 | 73.87 |
| 195 | Ghazipur | 1223 | 504 | 35.64 | 59.27 | 1,223 | 192 | 8.22 | 79.96 |
| 196 | Chandauli | 1328 | 415 | 38.26 | 19.5 | 1,328 | 85 | 5.85 | 41.71 |
| 197 | Varanasi | 2442 | 1246 | 58.46 | 30.3 | 2,442 | 319 | 11.73 | 47.59 |
| 198 | Sant ravidas nag | 1317 | 594 | 42.98 | 39.15 | 1,317 | 162 | 6.71 | 56.85 |
| 199 | Mirzapur | 1113 | 425 | 48.58 | 7.5 | 1,113 | 50 | 4.22 | 19.30 |
| 200 | Sonbhadra | 1027 | 386 | 44.5 | 15.24 | 1,027 | 65 | 6.17 | 25.76 |
| 201 | Etah | 1163 | 822 | 54.54 | 40.24 | 1,163 | 234 | 13.63 | 45.74 |
| 202 | Kanshiram nagar | 1146 | 1036 | 60.03 | 59.25 | 1,146 | 390 | 19.94 | 61.30 |
|  | **Bihar** |  |  |  |  |  |  |  |  |
| 203 | Pashchim champar | 952 | 53 | 4.02 | 48.24 | 952 | 36 | 1.89 | 63.73 |
| 204 | Purba champaran | 1072 | 68 | 5.54 | 39.01 | 1,072 | 37 | 1.92 | 75.39 |
| 205 | Sheohar | 1019 | 190 | 18.65 | 28.48 | 1,019 | 46 | 2.39 | 63.43 |
| 206 | Sitamarhi | 1118 | 396 | 33.79 | 36.25 | 1,118 | 100 | 5.12 | 60.75 |
| 207 | Madhubani | 1126 | 191 | 16.46 | 39.22 | 1,126 | 58 | 2 | 85.81 |
| 208 | Supaul | 1181 | 346 | 33.74 | 3.94 | 1,181 | 24 | 1.35 | 29.74 |
| 209 | Araria | 1133 | 303 | 29.85 | 26.91 | 1,133 | 58 | 2.87 | 63.59 |
| 210 | Kishanganj | 1183 | 143 | 12.18 | 38.55 | 1,183 | 60 | 3.1 | 59.48 |
| 211 | Purnia | 1114 | 336 | 31.58 | 28.97 | 1,114 | 79 | 2.61 | 79.01 |
| 212 | Katihar | 1001 | 232 | 26.64 | 11.51 | 1,001 | 29 | 1.75 | 50.97 |
| 213 | Madhepura | 1116 | 239 | 23.72 | 7.7 | 1,116 | 19 | 0.97 | 52.65 |
| 214 | Saharsa | 1139 | 296 | 29.23 | 17.13 | 1,139 | 48 | 2.46 | 54.76 |
| 215 | Darbhanga | 1126 | 172 | 17.43 | 12.45 | 1,126 | 18 | 1.66 | 7.96 |
| 216 | Muzaffarpur | 1084 | 108 | 9.22 | 26.91 | 1,084 | 32 | 0.72 | 90.05 |
| 217 | Gopalganj | 1286 | 114 | 9.04 | 32.35 | 1,286 | 41 | 1.66 | 64.79 |
| 218 | Siwan | 1350 | 151 | 9.84 | 43.57 | 1,350 | 74 | 2.79 | 61.52 |
| 219 | Saran | 1130 | 107 | 8.66 | 46.85 | 1,130 | 60 | 3.02 | 57.60 |
| 220 | Vaishali | 1242 | 299 | 23.97 | 33.78 | 1,242 | 61 | 1.88 | 72.70 |
| 221 | Samastipur | 1034 | 132 | 12.97 | 24.95 | 1,034 | 57 | 4.38 | 39.00 |
| 222 | Begusarai | 1203 | 358 | 35.24 | 8.89 | 1,203 | 34 | 1.93 | 54.66 |
| 223 | Khagaria | 1131 | 285 | 28.33 | 5.86 | 1,131 | 36 | 2.42 | 32.36 |
| 224 | Bhagalpur | 1203 | 250 | 24.63 | 14.07 | 1,203 | 41 | 2.2 | 60.49 |
| 225 | Banka | 1174 | 284 | 26.91 | 8.51 | 1,174 | 28 | 1.47 | 48.65 |
| 226 | Munger | 1045 | 336 | 35.44 | 19.05 | 1,045 | 50 | 2.93 | 62.40 |
| 227 | Lakhisarai | 1210 | 363 | 34.73 | 13.73 | 1,210 | 55 | 3.71 | 44.34 |
| 228 | Sheikhpura | 1222 | 355 | 32.56 | 20.02 | 1,222 | 64 | 3.48 | 53.99 |
| 229 | Nalanda | 1066 | 271 | 30.49 | 10.35 | 1,066 | 33 | 2.66 | 64.24 |
| 230 | Patna | 2441 | 790 | 39.38 | 15.17 | 2,441 | 142 | 4.75 | 36.21 |
| 231 | Bhojpur | 1397 | 296 | 27.74 | 9.38 | 1,397 | 41 | 3.27 | 32.23 |
| 232 | Buxar | 1303 | 375 | 34.98 | 15.42 | 1,303 | 69 | 3.5 | 47.59 |
| 233 | Kaimur (bhabua) | 1175 | 322 | 34.12 | 8.56 | 1,175 | 29 | 1.94 | 48.12 |
| 234 | Rohtas | 1390 | 482 | 44.75 | 9.2 | 1,390 | 39 | 2.05 | 62.34 |
| 235 | Aurangabad | 1194 | 322 | 32.82 | 10.25 | 1,194 | 35 | 2.1 | 59.64 |
| 236 | Gaya | 1443 | 439 | 35.66 | 8.46 | 1,443 | 49 | 2.31 | 48.35 |
| 237 | Nawada | 1228 | 333 | 30.63 | 17.98 | 1,228 | 66 | 2.98 | 46.94 |
| 238 | Jamui | 1199 | 267 | 23.72 | 17.19 | 1,199 | 42 | 1.83 | 56.12 |
| 239 | Jehanabad | 1107 | 348 | 34.9 | 20.64 | 1,107 | 69 | 4.24 | 45.90 |
| 240 | Arwal | 1275 | 308 | 29.03 | 16.15 | 1,275 | 44 | 2.46 | 56.88 |
|  | **Sikkim** |  |  |  |  |  |  |  |  |
| 241 | North district | 1049 | 379 | 50.21 | 13.43 | 1,049 | 243 | 31.31 | 15.52 |
| 242 | West district | 1125 | 539 | 65.84 | 13.79 | 1,125 | 336 | 37.28 | 14.71 |
| 243 | South district | 1091 | 459 | 59.25 | 19.58 | 1,091 | 224 | 25.44 | 24.43 |
| 244 | East district | 2028 | 499 | 32.12 | 24.57 | 2,028 | 318 | 18.64 | 26.73 |
|  | **Arunachal Pradesh** |  |  |  |  |  |  |  |  |
| 245 | Tawang | 689 | 141 | 22.27 | 38.9 | 689 | 120 | 17.75 | 40.27 |
| 246 | West kameng | 704 | 160 | 18.93 | 46.79 | 704 | 139 | 15.16 | 49.33 |
| 247 | East kameng | 783 | 65 | 5.15 | 54.64 | 783 | 57 | 4.19 | 56.24 |
| 248 | Papumpare | 1779 | 247 | 12.82 | 50.57 | 1,779 | 189 | 7.93 | 52.98 |
| 249 | Upper subansiri | 916 | 192 | 17.92 | 33.36 | 916 | 132 | 10.5 | 34.84 |
| 250 | West siang | 763 | 102 | 9.29 | 34.04 | 763 | 76 | 5.06 | 36.28 |
| 251 | East siang | 843 | 305 | 32.54 | 50.07 | 843 | 183 | 13.96 | 53.75 |
| 252 | Upper siang | 749 | 439 | 38.92 | 55.21 | 749 | 348 | 27.19 | 57.36 |
| 253 | Changlang | 945 | 781 | 62.56 | 44.58 | 945 | 331 | 25.35 | 36.95 |
| 254 | Tirap | 1046 | 768 | 57.76 | 45.23 | 1,046 | 349 | 25.03 | 39.14 |
| 255 | Lower subansiri | 734 | 78 | 10.57 | 30.53 | 734 | 43 | 5.12 | 33.67 |
| 256 | Kurung kumey | 902 | 161 | 12.87 | 41.55 | 902 | 109 | 6.4 | 48.72 |
| 257 | Dibang valley | 742 | 461 | 44.03 | 55.34 | 742 | 386 | 30.71 | 56.24 |
| 258 | Lower dibang val | 891 | 289 | 31 | 32.47 | 891 | 203 | 18.49 | 33.97 |
| 259 | Lohit | 1021 | 694 | 65.99 | 22.72 | 1,021 | 314 | 28.08 | 18.10 |
| 260 | Anjaw | 787 | 192 | 24.45 | 33.42 | 787 | 141 | 15.96 | 32.38 |
|  | **Nagaland** |  |  |  |  |  |  |  |  |
| 261 | Mon | 967 | 136 | 12.48 | 26.55 | 967 | 60 | 6.33 | 25.25 |
| 262 | Mokokchung | 644 | 183 | 38.24 | 13.67 | 644 | 63 | 10.04 | 28.29 |
| 263 | Zunheboto | 815 | 183 | 32.53 | 10.31 | 815 | 94 | 15.56 | 14.85 |
| 264 | Wokha | 637 | 159 | 30.74 | 20.94 | 637 | 50 | 8.57 | 36.20 |
| 265 | Dimapur | 1993 | 527 | 27.38 | 43.86 | 1,993 | 331 | 15.39 | 47.80 |
| 266 | Phek | 869 | 173 | 26.16 | 37.3 | 869 | 104 | 12.74 | 52.65 |
| 267 | Tuensang | 677 | 125 | 21.2 | 16.74 | 677 | 75 | 12.19 | 14.36 |
| 268 | Longleng | 786 | 137 | 20.94 | 8.29 | 786 | 45 | 6.49 | 15.62 |
| 269 | Kiphire | 806 | 289 | 31.13 | 39.76 | 806 | 106 | 13.01 | 38.88 |
| 270 | Kohima | 1787 | 390 | 33.75 | 25.31 | 1,787 | 154 | 12.51 | 22.55 |
| 271 | Peren | 809 | 291 | 30.95 | 48.4 | 809 | 182 | 17.38 | 50.45 |
|  | **Manipur** |  |  |  |  |  |  |  |  |
| 272 | Senapati (exclud | 1052 | 216 | 17.47 | 40.17 | 1,052 | 111 | 8.99 | 36.53 |
| 273 | Tamenglong | 964 | 191 | 16.22 | 44.34 | 964 | 89 | 6.68 | 46.05 |
| 274 | Churachandpur | 852 | 220 | 23.62 | 25.8 | 852 | 103 | 9.79 | 29.27 |
| 275 | Bishnupur | 2257 | 976 | 32.68 | 45.69 | 2,257 | 452 | 13.08 | 46.00 |
| 276 | Thoubal | 2302 | 717 | 23.99 | 42.98 | 2,302 | 370 | 11.04 | 44.24 |
| 277 | Imphal west | 2129 | 520 | 20.42 | 50.65 | 2,129 | 248 | 7.98 | 56.53 |
| 278 | Imphal east | 2232 | 887 | 30.9 | 35.46 | 2,232 | 346 | 10.55 | 41.84 |
| 279 | Ukhrul | 833 | 133 | 12.66 | 40.05 | 833 | 57 | 6.03 | 45.18 |
| 280 | Chandel | 972 | 168 | 15.35 | 36.06 | 972 | 77 | 6.4 | 39.19 |
|  | **Mizoram** |  |  |  |  |  |  |  |  |
| 281 | Mamit | 890 | 265 | 34.3 | 39.06 | 890 | 173 | 18.32 | 45.70 |
| 282 | Kolasib | 1660 | 529 | 33.51 | 29.37 | 1,660 | 355 | 17.87 | 33.51 |
| 283 | Aizawl | 1056 | 348 | 40.92 | 46.78 | 1,056 | 225 | 20.48 | 55.28 |
| 284 | Champhai | 1999 | 539 | 32.41 | 33.98 | 1,999 | 382 | 19.62 | 38.82 |
| 285 | Serchhip | 1770 | 642 | 41.16 | 34.71 | 1,770 | 422 | 23.79 | 38.35 |
| 286 | Lunglei | 1825 | 425 | 28.97 | 30.73 | 1,825 | 269 | 16.02 | 35.03 |
| 287 | Lawngtlai | 982 | 195 | 27.06 | 28.04 | 982 | 93 | 11.08 | 35.67 |
| 288 | Saiha | 2097 | 466 | 33.4 | 28.78 | 2,097 | 149 | 7.86 | 41.67 |
|  | **Tripura** |  |  |  |  |  |  |  |  |
| 289 | West tripura | 1938 | 1269 | 61.86 | 22.38 | 1,938 | 614 | 26.96 | 22.86 |
| 290 | South tripura | 937 | 658 | 66.94 | 18.13 | 937 | 325 | 32.96 | 16.00 |
| 291 | Dhalai | 968 | 705 | 67.1 | 20.83 | 968 | 331 | 29.66 | 17.70 |
| 292 | North tripura | 961 | 685 | 65.22 | 26.69 | 961 | 310 | 28.67 | 27.66 |
|  | **Meghalaya** |  |  |  |  |  |  |  |  |
| 293 | West garo hills | 1195 | 537 | 33.95 | 40.96 | 1,195 | 422 | 30.31 | 32.14 |
| 294 | East garo hills | 1235 | 168 | 12.79 | 48.08 | 1,235 | 150 | 11.04 | 49.54 |
| 295 | South garo hills | 1295 | 218 | 21.75 | 38.38 | 1,295 | 138 | 13.67 | 41.27 |
| 296 | West khasi hills | 1051 | 234 | 21.94 | 27.05 | 1,051 | 132 | 12.42 | 17.79 |
| 297 | Ribhoi | 1119 | 261 | 23.71 | 35.19 | 1,119 | 187 | 16.18 | 35.74 |
| 298 | East khasi hills | 2174 | 397 | 24.9 | 29.9 | 2,174 | 177 | 8.58 | 44.40 |
| 299 | Jaintia hills | 1133 | 235 | 20.9 | 27.54 | 1,133 | 145 | 10.78 | 31.54 |
|  | **Assam** |  |  |  |  |  |  |  |  |
| 300 | Kokrajhar | 980 | 902 | 56.67 | 46.51 | 980 | 504 | 33.11 | 40.61 |
| 301 | Dhubri | 994 | 535 | 34.21 | 48.94 | 994 | 405 | 25.55 | 48.02 |
| 302 | Goalpara | 1043 | 685 | 43.71 | 47.41 | 1,043 | 485 | 31.05 | 46.59 |
| 303 | Barpeta | 1092 | 802 | 56.73 | 43.66 | 1,092 | 560 | 36.73 | 46.82 |
| 304 | Morigaon | 1073 | 755 | 51.1 | 30.61 | 1,073 | 388 | 25.3 | 26.81 |
| 305 | Nagaon | 1006 | 628 | 53.71 | 26.92 | 1,006 | 421 | 35.41 | 26.07 |
| 306 | Sonitpur | 1073 | 728 | 64.11 | 25 | 1,073 | 402 | 36.39 | 20.49 |
| 307 | Lakhimpur | 1065 | 596 | 49.09 | 23.75 | 1,065 | 334 | 25.7 | 24.11 |
| 308 | Dhemaji | 1106 | 896 | 54.77 | 30.79 | 1,106 | 291 | 14.76 | 39.50 |
| 309 | Tinsukia | 1127 | 690 | 56.11 | 34.09 | 1,127 | 226 | 12.5 | 44.29 |
| 310 | Dibrugarh | 1093 | 668 | 53.82 | 34.89 | 1,093 | 282 | 16.59 | 44.77 |
| 311 | Sivasagar | 1054 | 690 | 50.01 | 30.68 | 1,054 | 226 | 15.28 | 32.34 |
| 312 | Jorhat | 1112 | 614 | 53.04 | 18.81 | 1,112 | 243 | 20.01 | 18.32 |
| 313 | Golaghat | 1089 | 579 | 45.96 | 28.97 | 1,089 | 317 | 23.43 | 29.55 |
| 314 | Karbi anglong | 1014 | 678 | 53.32 | 26.04 | 1,014 | 450 | 36.81 | 22.20 |
| 315 | Dima hasao | 984 | 603 | 48.74 | 26.97 | 984 | 292 | 26.49 | 18.97 |
| 316 | Cachar | 1044 | 638 | 54.41 | 38.68 | 1,044 | 214 | 15.02 | 47.13 |
| 317 | Karimganj | 1141 | 650 | 52.1 | 26.17 | 1,141 | 389 | 28.7 | 27.92 |
| 318 | Hailakandi | 1073 | 521 | 54.17 | 15.94 | 1,073 | 288 | 28.32 | 17.58 |
| 319 | Bongaigaon | 1005 | 786 | 59.42 | 29.52 | 1,005 | 461 | 35.17 | 24.61 |
| 320 | Chirang | 996 | 746 | 62.6 | 34.22 | 996 | 381 | 29.45 | 39.10 |
| 321 | Kamrup | 1085 | 500 | 35.86 | 43.82 | 1,085 | 331 | 21.71 | 46.76 |
| 322 | Kamrup metropoli | 881 | 519 | 46.28 | 40.21 | 881 | 292 | 23.77 | 40.70 |
| 323 | Nalbari | 1103 | 808 | 63.24 | 32.86 | 1,103 | 478 | 37.13 | 32.18 |
| 324 | Baksa | 1044 | 937 | 58.09 | 47.33 | 1,044 | 510 | 30.46 | 41.55 |
| 325 | Darrang | 1087 | 844 | 65.73 | 34.43 | 1,087 | 534 | 41.74 | 29.61 |
| 326 | Udalguri | 1083 | 764 | 63.85 | 22.44 | 1,083 | 439 | 36.76 | 18.15 |
|  | **West Bengal** |  |  |  |  |  |  |  |  |
| 327 | Darjiling | 928 | 588 | 73.35 | 26.91 | 928 | 252 | 26.51 | 31.39 |
| 328 | Jalpaiguri | 899 | 462 | 49.06 | 34.65 | 899 | 256 | 22.03 | 37.93 |
| 329 | Koch bihar | 886 | 753 | 65.66 | 50.27 | 886 | 361 | 25.49 | 52.42 |
| 330 | Uttar dinajpur | 1012 | 724 | 54.74 | 46.31 | 1,012 | 333 | 24.34 | 49.52 |
| 331 | Dakshin dinajpur | 867 | 620 | 60.28 | 32.61 | 867 | 358 | 31.98 | 30.43 |
| 332 | Maldah | 911 | 716 | 60.27 | 42.06 | 911 | 358 | 25.96 | 42.78 |
| 333 | Murshidabad | 1006 | 983 | 72.79 | 37.19 | 1,006 | 374 | 20.26 | 39.21 |
| 334 | Birbhum | 950 | 893 | 77.12 | 38.13 | 950 | 396 | 27.99 | 35.54 |
| 335 | Barddhaman | 986 | 854 | 77.11 | 32.39 | 986 | 305 | 22 | 34.40 |
| 336 | Nadia | 928 | 813 | 74.31 | 27.41 | 928 | 273 | 18.93 | 30.86 |
| 337 | North twenty fou | 872 | 943 | 72.98 | 46.45 | 872 | 455 | 30.35 | 44.18 |
| 338 | Hugli | 871 | 801 | 77.92 | 36.02 | 871 | 326 | 22.81 | 44.93 |
| 339 | Bankura | 939 | 759 | 76.25 | 23.13 | 939 | 327 | 26.22 | 28.19 |
| 340 | Puruliya | 1012 | 822 | 68.97 | 31.61 | 1,012 | 191 | 12.36 | 37.23 |
| 341 | Haora | 930 | 792 | 67.5 | 33.3 | 930 | 380 | 29.99 | 31.75 |
| 342 | Kolkata | 803 | 617 | 69.98 | 36.88 | 803 | 336 | 38.46 | 36.43 |
| 343 | South twenty fou | 930 | 860 | 68.62 | 37.1 | 930 | 586 | 44.24 | 32.70 |
| 344 | Paschim medinipu | 1013 | 870 | 73.8 | 36.38 | 1,013 | 356 | 23.14 | 37.25 |
| 345 | Purba medinipur | 925 | 972 | 75.26 | 34.24 | 925 | 574 | 39.79 | 31.79 |
|  | **Jharkhand** |  |  |  |  |  |  |  |  |
| 346 | Garhwa | 985 | 314 | 34.05 | 31.43 | 985 | 49 | 1.76 | 73.49 |
| 347 | Chatra | 1040 | 340 | 36.18 | 19.19 | 1,040 | 47 | 2.26 | 54.80 |
| 348 | Kodarma | 1083 | 594 | 62.21 | 9.1 | 1,083 | 70 | 6.97 | 21.52 |
| 349 | Giridih | 1119 | 533 | 47.74 | 21.65 | 1,119 | 83 | 3.49 | 56.65 |
| 350 | Deoghar | 1064 | 444 | 46.05 | 10 | 1,064 | 76 | 5.11 | 33.66 |
| 351 | Godda | 850 | 359 | 46.19 | 14.67 | 850 | 57 | 5.8 | 31.64 |
| 352 | Sahibganj | 979 | 300 | 34.59 | 12.72 | 979 | 92 | 7.48 | 28.20 |
| 353 | Pakur | 1059 | 327 | 35 | 21.91 | 1,059 | 57 | 3.56 | 66.63 |
| 354 | Dhanbad | 2181 | 913 | 49.77 | 21.73 | 2,181 | 265 | 9.87 | 39.87 |
| 355 | Bokaro | 2092 | 898 | 54.44 | 12.16 | 2,092 | 119 | 5.05 | 39.08 |
| 356 | Lohardaga | 962 | 192 | 24.31 | 29.71 | 962 | 64 | 5.05 | 49.86 |
| 357 | Purbi singhbhum | 1952 | 472 | 32.89 | 13.31 | 1,952 | 142 | 8.47 | 25.62 |
| 358 | Palamu | 928 | 210 | 26.07 | 22.32 | 928 | 35 | 2.44 | 63.13 |
| 359 | Latehar | 953 | 199 | 25.99 | 7.74 | 953 | 30 | 3.1 | 24.57 |
| 360 | Hazaribagh | 1142 | 563 | 55.76 | 19.9 | 1,142 | 87 | 4.62 | 44.55 |
| 361 | Ramgarh | 2100 | 798 | 45.23 | 19.36 | 2,100 | 156 | 5.44 | 41.56 |
| 362 | Dumka | 951 | 319 | 36.33 | 18.68 | 951 | 72 | 6.32 | 29.67 |
| 363 | Jamtara | 1094 | 448 | 44.02 | 17.55 | 1,094 | 156 | 13.35 | 25.62 |
| 364 | Ranchi | 1761 | 623 | 44.25 | 19.93 | 1,761 | 202 | 10.63 | 34.91 |
| 365 | Khunti | 996 | 286 | 37.55 | 11.22 | 996 | 119 | 14.11 | 17.95 |
| 366 | Gumla | 1045 | 228 | 26 | 31 | 1,045 | 75 | 6.65 | 48.65 |
| 367 | Simdega | 881 | 193 | 25.66 | 27.98 | 881 | 57 | 5.65 | 39.92 |
| 368 | Pashchimi singhb | 939 | 126 | 15.93 | 24.22 | 939 | 37 | 2.62 | 48.88 |
| 369 | Saraikela kharsa | 890 | 218 | 28.38 | 31.33 | 890 | 72 | 7.73 | 53.11 |
|  | **Odisha** |  |  |  |  |  |  |  |  |
| 370 | Bargarh | 1007 | 712 | 67.07 | 31.19 | 1,007 | 226 | 17.79 | 31.21 |
| 371 | Jharsuguda | 2189 | 1534 | 73.98 | 29.87 | 2,189 | 639 | 24.55 | 33.99 |
| 372 | Sambalpur | 963 | 686 | 60.34 | 45.75 | 963 | 247 | 15.54 | 45.09 |
| 373 | Debagarh | 917 | 493 | 44.74 | 47.3 | 917 | 186 | 13.11 | 60.91 |
| 374 | Sundargarh | 2175 | 1481 | 63.29 | 44.37 | 2,175 | 423 | 15.7 | 34.02 |
| 375 | Kendujhar | 973 | 610 | 39.81 | 59.86 | 973 | 342 | 18.3 | 63.41 |
| 376 | Mayurbhanj | 957 | 590 | 41.15 | 51.71 | 957 | 281 | 15.27 | 59.85 |
| 377 | Baleshwar | 1012 | 531 | 38.45 | 47.35 | 1,012 | 325 | 18.57 | 50.18 |
| 378 | Bhadrak | 1017 | 353 | 29.32 | 53.6 | 1,017 | 155 | 8.16 | 63.21 |
| 379 | Kendrapara | 1021 | 772 | 62.84 | 42.67 | 1,021 | 395 | 26.3 | 41.65 |
| 380 | Jagatsinghapur | 1049 | 767 | 63.82 | 40.05 | 1,049 | 294 | 17.8 | 49.76 |
| 381 | Cuttack | 876 | 458 | 49.78 | 31.43 | 876 | 140 | 10.51 | 38.19 |
| 382 | Jajapur | 1085 | 1068 | 58.85 | 64.9 | 1,085 | 476 | 18.98 | 66.67 |
| 383 | Dhenkanal | 1000 | 796 | 69.19 | 44.77 | 1,000 | 246 | 16 | 49.45 |
| 384 | Anugul | 1135 | 747 | 67 | 26.96 | 1,135 | 281 | 23.76 | 21.93 |
| 385 | Nayagarh | 975 | 1198 | 62.9 | 64.62 | 975 | 445 | 19.34 | 61.62 |
| 386 | Khordha | 1822 | 1404 | 64.38 | 33.13 | 1,822 | 600 | 23.18 | 37.36 |
| 387 | Puri | 1135 | 973 | 70.47 | 44.4 | 1,135 | 386 | 19.51 | 47.78 |
| 388 | Ganjam | 948 | 707 | 59.05 | 51.32 | 948 | 310 | 16.34 | 50.69 |
| 389 | Gajapati | 978 | 688 | 65.77 | 44.21 | 978 | 170 | 9.94 | 45.44 |
| 390 | Kandhamal | 1133 | 830 | 60.36 | 45.19 | 1,133 | 359 | 19.7 | 53.02 |
| 391 | Baudh | 1050 | 666 | 64.52 | 22.26 | 1,050 | 276 | 22.71 | 25.10 |
| 392 | Subarnapur | 1067 | 881 | 60.7 | 58.07 | 1,067 | 325 | 19.69 | 49.66 |
| 393 | Balangir | 1047 | 690 | 65.25 | 26.65 | 1,047 | 249 | 17.59 | 30.53 |
| 394 | Nuapada | 1092 | 678 | 58.8 | 25.24 | 1,092 | 209 | 12.55 | 28.99 |
| 395 | Kalahandi | 892 | 716 | 61.55 | 44.07 | 892 | 228 | 15.96 | 48.48 |
| 396 | Rayagada | 1030 | 861 | 54.91 | 60.15 | 1,030 | 212 | 10.89 | 45.78 |
| 397 | Nabarangapur | 1191 | 948 | 64.44 | 42.08 | 1,191 | 314 | 15.1 | 46.32 |
| 398 | Koraput | 963 | 677 | 59.21 | 46.64 | 963 | 207 | 11.98 | 47.85 |
| 399 | Malkangiri | 1022 | 858 | 51.97 | 55.41 | 1,022 | 232 | 11.43 | 52.48 |
|  | **Chhattisgarh** |  |  |  |  |  |  |  |  |
| 400 | Korea (koriya) | 2131 | 1068 | 50.56 | 34.6 | 2,131 | 440 | 12.95 | 48.38 |
| 401 | Surguja | 938 | 342 | 41.41 | 20.59 | 938 | 68 | 4.73 | 41.87 |
| 402 | Jashpur | 924 | 404 | 50.12 | 14.38 | 924 | 83 | 7.47 | 35.46 |
| 403 | Raigarh | 967 | 459 | 52.29 | 17.66 | 967 | 112 | 6.61 | 63.32 |
| 404 | Korba | 2378 | 1180 | 55.45 | 20.51 | 2,378 | 333 | 10.5 | 33.81 |
| 405 | Janjgir - champa | 1134 | 486 | 61.74 | 14.2 | 1,134 | 69 | 6.19 | 37.90 |
| 406 | Bilaspur | 1232 | 976 | 64.59 | 48.84 | 1,232 | 474 | 16.01 | 57.49 |
| 407 | Kabirdham | 1126 | 504 | 58.05 | 12.63 | 1,126 | 43 | 2.68 | 47.75 |
| 408 | Rajnandgaon | 1309 | 931 | 64.26 | 48.84 | 1,309 | 380 | 11.71 | 61.74 |
| 409 | Durg | 2269 | 1187 | 66.6 | 19.5 | 2,269 | 203 | 6.6 | 46.46 |
| 410 | Raipur | 2251 | 1022 | 57.47 | 16.84 | 2,251 | 165 | 5.7 | 37.83 |
| 411 | Mahasamund | 1130 | 618 | 65.54 | 19.9 | 1,130 | 79 | 4.5 | 47.51 |
| 412 | Dhamtari | 1251 | 705 | 72 | 17.45 | 1,251 | 78 | 3.82 | 38.31 |
| 413 | Uttar bastar kan | 1132 | 448 | 55.85 | 5.05 | 1,132 | 42 | 4.66 | 22.50 |
| 414 | Bastar | 1153 | 440 | 45.75 | 19.18 | 1,153 | 75 | 5.41 | 34.40 |
| 415 | Narayanpur | 1434 | 521 | 40.03 | 34.69 | 1,434 | 202 | 8.73 | 48.76 |
| 416 | Dakshin bastar d | 1144 | 359 | 39.42 | 17.76 | 1,144 | 46 | 3.41 | 52.98 |
| 417 | Bijapur | 1269 | 683 | 51.44 | 46.02 | 1,269 | 247 | 8.41 | 60.68 |
|  | **Madhya Pradesh** |  |  |  |  |  |  |  |  |
| 418 | Sheopur | 1098 | 465 | 53.24 | 11.54 | 1,098 | 63 | 4.58 | 43.33 |
| 419 | Morena | 1125 | 561 | 56.3 | 11.03 | 1,125 | 96 | 6.05 | 29.29 |
| 420 | Bhind | 1049 | 551 | 55.7 | 30.4 | 1,049 | 126 | 8.29 | 58.41 |
| 421 | Gwalior | 2325 | 962 | 49.17 | 29.87 | 2,325 | 306 | 12.9 | 46.13 |
| 422 | Datia | 1121 | 576 | 58.99 | 16.41 | 1,121 | 98 | 6.1 | 45.06 |
| 423 | Shivpuri | 1132 | 575 | 57.69 | 18.78 | 1,132 | 86 | 4.96 | 51.56 |
| 424 | Tikamgarh | 1060 | 469 | 48.5 | 27.98 | 1,060 | 62 | 3.86 | 60.63 |
| 425 | Chhatarpur | 1000 | 498 | 53.93 | 25.22 | 1,000 | 83 | 4.68 | 61.40 |
| 426 | Panna | 954 | 365 | 45.92 | 19.46 | 954 | 46 | 3.27 | 43.60 |
| 427 | Sagar | 960 | 449 | 48.95 | 27.52 | 960 | 86 | 5.35 | 59.48 |
| 428 | Damoh | 1015 | 318 | 36.23 | 23.17 | 1,015 | 53 | 2.85 | 66.79 |
| 429 | Satna | 1010 | 459 | 54.93 | 21.72 | 1,010 | 66 | 5.23 | 44.32 |
| 430 | Rewa | 1092 | 543 | 59.26 | 29.32 | 1,092 | 58 | 3.87 | 58.30 |
| 431 | Umaria | 973 | 437 | 52.29 | 20.06 | 973 | 47 | 3.2 | 63.42 |
| 432 | Neemuch | 1186 | 199 | 17.64 | 34.38 | 1,186 | 70 | 3.44 | 57.07 |
| 433 | Mandsaur | 1210 | 242 | 18.1 | 52.87 | 1,210 | 117 | 5.03 | 70.62 |
| 434 | Ratlam | 1088 | 297 | 24.75 | 36.79 | 1,088 | 95 | 4.07 | 64.42 |
| 435 | Ujjain | 2489 | 877 | 35.06 | 26.38 | 2,489 | 329 | 8.3 | 42.08 |
| 436 | Shajapur | 1098 | 540 | 55.5 | 14.77 | 1,098 | 123 | 10.38 | 24.27 |
| 437 | Dewas | 1136 | 602 | 56.59 | 28.04 | 1,136 | 175 | 9.02 | 51.15 |
| 438 | Dhar | 1237 | 592 | 52.57 | 23.34 | 1,237 | 143 | 7.46 | 48.10 |
| 439 | Indore | 2412 | 1064 | 54.04 | 16.38 | 2,412 | 376 | 15.17 | 26.23 |
| 440 | Khargone (west n | 1333 | 806 | 70.57 | 29.74 | 1,333 | 168 | 7.15 | 57.26 |
| 441 | Barwani | 1332 | 600 | 50.7 | 26.58 | 1,332 | 117 | 4.97 | 68.60 |
| 442 | Rajgarh | 1025 | 460 | 53.52 | 17.66 | 1,025 | 101 | 9.14 | 42.85 |
| 443 | Vidisha | 937 | 223 | 22.97 | 45.84 | 937 | 95 | 6.63 | 68.67 |
| 444 | Bhopal | 1104 | 456 | 52.91 | 16.68 | 1,104 | 168 | 16.92 | 24.35 |
| 445 | Sehore | 1104 | 576 | 55.07 | 32.49 | 1,104 | 190 | 9.54 | 52.91 |
| 446 | Raisen | 1171 | 643 | 66 | 18.17 | 1,171 | 152 | 11.78 | 36.70 |
| 447 | Betul | 1067 | 546 | 63.97 | 14.83 | 1,067 | 86 | 6.58 | 35.96 |
| 448 | Harda | 1202 | 520 | 49.4 | 22.9 | 1,202 | 125 | 8.23 | 45.18 |
| 449 | Hoshangabad | 2435 | 1003 | 50.18 | 29.93 | 2,435 | 238 | 7.07 | 54.61 |
| 450 | Katni | 1072 | 389 | 45.73 | 16.56 | 1,072 | 46 | 2.84 | 61.98 |
| 451 | Jabalpur | 2106 | 1134 | 65.91 | 17.94 | 2,106 | 160 | 7.04 | 41.82 |
| 452 | Narsimhapur | 1091 | 457 | 51.03 | 17.57 | 1,091 | 44 | 3.22 | 53.52 |
| 453 | Dindori | 1039 | 583 | 66.81 | 16.9 | 1,039 | 21 | 0.88 | 77.28 |
| 454 | Mandla | 982 | 548 | 66.9 | 14.36 | 982 | 34 | 2.55 | 56.79 |
| 455 | Chhindwara | 1157 | 590 | 66.06 | 9.12 | 1,157 | 62 | 5.05 | 33.53 |
| 456 | Seoni | 1109 | 575 | 70 | 6.12 | 1,109 | 31 | 3 | 41.50 |
| 457 | Balaghat | 1074 | 473 | 58.18 | 10.48 | 1,074 | 48 | 3.56 | 51.18 |
| 458 | Guna | 1256 | 673 | 60.9 | 17.74 | 1,256 | 162 | 10.53 | 33.50 |
| 459 | Ashoknagar | 1083 | 637 | 58.29 | 35.87 | 1,083 | 189 | 9.09 | 56.45 |
| 460 | Shahdol | 975 | 344 | 43.45 | 12.37 | 975 | 38 | 2.48 | 70.09 |
| 461 | Anuppur | 1102 | 420 | 49.06 | 10.75 | 1,102 | 44 | 3.64 | 43.66 |
| 462 | Sidhi | 1068 | 286 | 32.51 | 10.28 | 1,068 | 25 | 1.58 | 43.42 |
| 463 | Singrauli | 1221 | 362 | 37.06 | 8.66 | 1,221 | 33 | 2.65 | 39.56 |
| 464 | Jhabua | 1001 | 124 | 10.36 | 39.91 | 1,001 | 50 | 1.95 | 67.49 |
| 465 | Alirajpur | 1430 | 434 | 30.93 | 43.36 | 1,430 | 145 | 4.15 | 74.16 |
| 466 | Khandwa (east ni | 1250 | 701 | 67.34 | 13.98 | 1,250 | 136 | 8.43 | 35.03 |
| 467 | Burhanpur | 2307 | 1107 | 56.43 | 22.07 | 2,307 | 316 | 10.23 | 45.27 |
|  | **Gujarat** |  |  |  |  |  |  |  |  |
| 468 | Kachchh | 837 | 286 | 37.74 | 33.17 | 837 | 74 | 6.34 | 56.73 |
| 469 | Banaskantha | 918 | 376 | 42.39 | 41.66 | 918 | 135 | 10.58 | 50.48 |
| 470 | Patan | 896 | 348 | 41.87 | 32.43 | 896 | 100 | 8.48 | 54.90 |
| 471 | Mahesana | 862 | 409 | 49.01 | 31.16 | 862 | 148 | 12.3 | 41.11 |
| 472 | Sabarkantha | 910 | 378 | 46.53 | 25.7 | 910 | 66 | 6.29 | 45.51 |
| 473 | Gandhinagar | 828 | 615 | 59.71 | 42.76 | 828 | 165 | 11.02 | 43.99 |
| 474 | Ahmadabad | 768 | 539 | 60.17 | 33.96 | 768 | 191 | 16.29 | 35.93 |
| 475 | Surendranagar | 864 | 438 | 56.91 | 29.48 | 864 | 107 | 10.94 | 38.17 |
| 476 | Rajkot | 880 | 371 | 44.48 | 27.01 | 880 | 136 | 14.22 | 32.79 |
| 477 | Jamnagar | 895 | 270 | 35.75 | 26.43 | 895 | 96 | 9.8 | 43.94 |
| 478 | Porbandar | 932 | 321 | 35.46 | 33.68 | 932 | 124 | 10.38 | 42.87 |
| 479 | Junagadh | 921 | 421 | 55.92 | 29.72 | 921 | 119 | 11.12 | 31.87 |
| 480 | Amreli | 933 | 299 | 37.63 | 33.03 | 933 | 67 | 3.9 | 55.91 |
| 481 | Bhavnagar | 926 | 297 | 34.42 | 34.9 | 926 | 95 | 5.74 | 55.14 |
| 482 | Anand | 898 | 526 | 51.46 | 46.2 | 898 | 119 | 5.9 | 56.48 |
| 483 | Kheda | 910 | 292 | 32.72 | 35.89 | 910 | 87 | 4.44 | 60.58 |
| 484 | Panchmahal | 935 | 218 | 24 | 30.02 | 935 | 38 | 2.29 | 62.79 |
| 485 | Dohad | 852 | 237 | 30.96 | 39.3 | 852 | 50 | 2.95 | 77.33 |
| 486 | Vadodara | 812 | 365 | 46.34 | 33.29 | 812 | 80 | 5.78 | 62.94 |
| 487 | Narmada | 935 | 471 | 54.95 | 30.93 | 935 | 74 | 4.25 | 51.32 |
| 488 | Bharuch | 832 | 657 | 68.19 | 40.03 | 832 | 143 | 8.62 | 46.91 |
| 489 | The dangs | 854 | 292 | 38.63 | 14.15 | 854 | 31 | 1.82 | 55.95 |
| 490 | Navsari | 852 | 561 | 68.73 | 31.66 | 852 | 111 | 8.01 | 32.71 |
| 491 | Valsad | 866 | 329 | 38.35 | 43.06 | 866 | 112 | 6.32 | 63.50 |
| 492 | Surat | 880 | 413 | 48.87 | 37.11 | 880 | 155 | 12.5 | 50.59 |
| 493 | Tapi | 936 | 395 | 51.32 | 23.37 | 936 | 52 | 2.63 | 54.35 |
|  | **Daman & Diu** |  |  |  |  |  |  |  |  |
| 494 | Diu | 806 | 266 | 45.88 | 27.33 | 806 | 63 | 5.34 | 41.26 |
| 495 | Daman | 587 | 151 | 29.04 | 19.6 | 587 | 44 | 5.94 | 34.84 |
|  | **Dadra & Nagar Haveli** |  |  |  |  |  |  |  |  |
| 496 | Dadra & Nagar Haveli | 796 | 254 | 38.03 | 13.9 | 796 | 57 | 6.22 | 29.91 |
|  | **Maharashtra** |  |  |  |  |  |  |  |  |
| 497 | Nandurbar | 900 | 415 | 55.28 | 18.85 | 900 | 60 | 4.33 | 45.33 |
| 498 | Dhule | 875 | 489 | 63.8 | 11.4 | 875 | 94 | 7.5 | 25.78 |
| 499 | Jalgaon | 799 | 531 | 65.29 | 32.58 | 799 | 161 | 11.03 | 45.63 |
| 500 | Buldana | 837 | 584 | 73.43 | 20.3 | 837 | 203 | 18.39 | 32.96 |
| 501 | Akola | 1029 | 598 | 67.43 | 21.21 | 1,029 | 195 | 13.49 | 36.23 |
| 502 | Washim | 876 | 579 | 75.52 | 8.36 | 876 | 126 | 12.31 | 15.34 |
| 503 | Amravati | 889 | 538 | 72.44 | 12.62 | 889 | 137 | 12.33 | 21.10 |
| 504 | Wardha | 856 | 575 | 78.2 | 16.47 | 856 | 125 | 8.38 | 30.92 |
| 505 | Nagpur | 1116 | 639 | 69.07 | 12.71 | 1,116 | 166 | 15.34 | 20.48 |
| 506 | Bhandara | 823 | 539 | 77.39 | 21.16 | 823 | 86 | 7.09 | 36.01 |
| 507 | Gondiya | 822 | 453 | 67.67 | 8.13 | 822 | 40 | 3.79 | 43.54 |
| 508 | Gadchiroli | 840 | 504 | 75.11 | 10.05 | 840 | 61 | 5.38 | 28.19 |
| 509 | Chandrapur | 819 | 503 | 73.17 | 9.42 | 819 | 78 | 6.37 | 22.76 |
| 510 | Yavatmal | 893 | 542 | 71.64 | 15.72 | 893 | 102 | 9.38 | 34.60 |
| 511 | Nanded | 978 | 544 | 60.92 | 16.56 | 978 | 96 | 5.26 | 43.97 |
| 512 | Hingoli | 872 | 578 | 66.86 | 17.78 | 872 | 130 | 8.68 | 28.12 |
| 513 | Parbhani | 916 | 622 | 69.62 | 22.72 | 916 | 206 | 11.91 | 38.66 |
| 514 | Jalna | 859 | 544 | 64.99 | 19.97 | 859 | 158 | 13.29 | 32.40 |
| 515 | Aurangabad | 860 | 602 | 64.62 | 29.04 | 860 | 267 | 20.85 | 34.24 |
| 516 | Nashik | 951 | 600 | 67.5 | 20.1 | 951 | 180 | 14.38 | 31.45 |
| 517 | Thane | 763 | 456 | 65.49 | 26.58 | 763 | 147 | 13.49 | 42.23 |
| 518 | Mumbai suburban | 673 | 376 | 64.93 | 24.81 | 673 | 136 | 16.04 | 38.45 |
| 519 | Mumbai | 608 | 335 | 59.61 | 32.28 | 608 | 143 | 16.95 | 42.72 |
| 520 | Raigarh | 759 | 461 | 63.57 | 17.66 | 759 | 144 | 13.27 | 31.97 |
| 521 | Pune | 742 | 537 | 70.62 | 32.98 | 742 | 179 | 14.28 | 46.25 |
| 522 | Ahmadnagar | 798 | 432 | 48.4 | 39.3 | 798 | 144 | 7.26 | 59.60 |
| 523 | Bid | 823 | 516 | 67.12 | 16.72 | 823 | 119 | 11.04 | 33.99 |
| 524 | Latur | 967 | 575 | 67.42 | 17.49 | 967 | 117 | 7.31 | 38.68 |
| 525 | Osmanabad | 769 | 504 | 70.31 | 21.32 | 769 | 107 | 9.33 | 39.77 |
| 526 | Solapur | 931 | 564 | 64.42 | 20.35 | 931 | 94 | 4.85 | 46.51 |
| 527 | Satara | 740 | 433 | 62.46 | 21.83 | 740 | 77 | 4.99 | 46.23 |
| 528 | Ratnagiri | 727 | 215 | 38.26 | 20.19 | 727 | 40 | 4.06 | 40.73 |
| 529 | Sindhudurg | 660 | 288 | 49.97 | 28.68 | 660 | 69 | 5.63 | 52.25 |
| 530 | Kolhapur | 827 | 404 | 53.68 | 24.66 | 827 | 95 | 5.58 | 46.26 |
| 531 | Sangli | 863 | 396 | 52.04 | 16.34 | 863 | 81 | 6.37 | 32.93 |
|  | **Andhra Pradesh** |  |  |  |  |  |  |  |  |
| 532 | Adilabad | 764 | 310 | 48.65 | 9.38 | 764 | 14 | 1.17 | 67.82 |
| 533 | Nizamabad | 752 | 287 | 47.01 | 2.99 | 752 | 4 | 0.16 | 63.24 |
| 534 | Karimnagar | 728 | 229 | 38.55 | 15.18 | 728 | 4 | 0.14 | 100.00 |
| 535 | Medak | 743 | 309 | 49.63 | 10.75 | 743 | 18 | 1.79 | 58.53 |
| 536 | Hyderabad | 830 | 349 | 56.46 | 7.77 | 830 | 20 | 1.96 | 45.22 |
| 537 | Rangareddy | 762 | 430 | 69.07 | 7.2 | 762 | 17 | 1.62 | 55.91 |
| 538 | Mahbubnagar | 757 | 417 | 64.32 | 4.97 | 757 | 8 | 0.3 | 70.98 |
| 539 | Nalgonda | 723 | 427 | 68.03 | 5.22 | 723 | 13 | 1.34 | 56.76 |
| 540 | Warangal | 730 | 306 | 50.81 | 3.46 | 730 | 12 | 1.74 | 23.32 |
| 541 | Khammam | 778 | 452 | 69.08 | 10.95 | 778 | 14 | 0.9 | 77.93 |
| 542 | Srikakulam | 815 | 473 | 67.69 | 7.55 | 815 | 6 | 0.69 | 47.71 |
| 543 | Vizianagaram | 850 | 504 | 71.58 | 1.83 | 850 | 4 | 0.16 | 65.86 |
| 544 | Visakhapatnam | 772 | 430 | 69.15 | 2.51 | 772 | 6 | 0.6 | 48.43 |
| 545 | East godavari | 802 | 489 | 72.17 | 7.25 | 802 | 10 | 0.49 | 78.07 |
| 546 | West godavari | 736 | 469 | 76.65 | 3.19 | 736 | 7 | 0.92 | 51.89 |
| 547 | Krishna | 836 | 516 | 74.79 | 3.81 | 836 | 11 | 0.78 | 64.77 |
| 548 | Guntur | 761 | 485 | 73.97 | 2.37 | 761 | 8 | 0.58 | 74.65 |
| 549 | Prakasam | 785 | 497 | 70.91 | 1.65 | 785 | 8 | 0.65 | 37.07 |
| 550 | Sri potti sriram | 855 | 514 | 71.03 | 5.46 | 855 | 7 | 0.35 | 100.00 |
| 551 | Y.s.r. | 782 | 389 | 59.36 | 5.7 | 782 | 8 | 0.4 | 78.02 |
| 552 | Kurnool | 899 | 512 | 65.92 | 0.75 | 899 | 4 | 0 | 25.64 |
| 553 | Anantapur | 795 | 436 | 65.3 | 4.56 | 795 | 8 | 0.31 | 75.24 |
| 554 | Chittoor | 740 | 372 | 59.67 | 1.2 | 740 | 7 | 1.22 | 18.59 |
|  | **Karnataka** |  |  |  |  |  |  |  |  |
| 555 | Belgaum | 930 | 479 | 60 | 12.7 | 930 | 27 | 1.77 | 62.70 |
| 556 | Bagalkot | 956 | 417 | 54.85 | 3.01 | 956 | 9 | 0.59 | 63.78 |
| 557 | Bijapur | 941 | 458 | 58.72 | 1.92 | 941 | 15 | 1.34 | 21.44 |
| 558 | Bidar | 993 | 442 | 59.89 | 6.45 | 993 | 17 | 2.21 | 49.22 |
| 559 | Raichur | 965 | 417 | 54.3 | 5.56 | 965 | 12 | 1.03 | 53.82 |
| 560 | Koppal | 917 | 344 | 44.45 | 4.55 | 917 | 7 | 0.13 | 47.58 |
| 561 | Gadag | 939 | 459 | 59.62 | 19.81 | 939 | 50 | 1.48 | 69.27 |
| 562 | Dharwad | 912 | 453 | 59.33 | 8.17 | 912 | 34 | 3.75 | 37.76 |
| 563 | Uttara kannada | 858 | 220 | 31.34 | 33.69 | 858 | 58 | 4.84 | 58.58 |
| 564 | Haveri | 856 | 335 | 48.59 | 9.98 | 856 | 9 | 0.17 | 87.57 |
| 565 | Bellary | 912 | 394 | 50.82 | 6.22 | 912 | 18 | 1.26 | 48.10 |
| 566 | Chitradurga | 937 | 494 | 64.08 | 27.97 | 937 | 44 | 2.87 | 59.20 |
| 567 | Davanagere | 874 | 407 | 57.69 | 13.23 | 874 | 35 | 2.14 | 61.09 |
| 568 | Shimoga | 863 | 268 | 36.84 | 33.79 | 863 | 57 | 3.61 | 63.74 |
| 569 | Udupi | 860 | 243 | 32.35 | 41.33 | 860 | 72 | 4.63 | 63.93 |
| 570 | Chikmagalur | 736 | 282 | 45.87 | 18.74 | 736 | 34 | 3.4 | 54.23 |
| 571 | Tumkur | 810 | 439 | 62.71 | 16.62 | 810 | 37 | 2.58 | 54.20 |
| 572 | Bangalore | 798 | 330 | 45.32 | 29.25 | 798 | 66 | 5.25 | 60.86 |
| 573 | Mandya | 798 | 386 | 59.39 | 2.6 | 798 | 7 | 0.5 | 31.50 |
| 574 | Hassan | 821 | 409 | 56.67 | 29.62 | 821 | 65 | 5.1 | 63.57 |
| 575 | Dakshina kannada | 859 | 151 | 20.97 | 36.45 | 859 | 41 | 2.58 | 56.76 |
| 576 | Kodagu | 804 | 285 | 41.94 | 18.45 | 804 | 28 | 2.54 | 67.21 |
| 577 | Mysore | 863 | 455 | 55.59 | 47.55 | 863 | 97 | 3.05 | 81.94 |
| 578 | Chamarajanagar | 859 | 382 | 51.68 | 10.41 | 859 | 26 | 2.05 | 46.44 |
| 579 | Gulbarga | 922 | 373 | 53.67 | 1.81 | 922 | 4 | 0.27 | 62.34 |
| 580 | Yadgir | 943 | 354 | 47 | 3.95 | 943 | 5 | 0 | 100.00 |
| 581 | Kolar | 906 | 445 | 62.67 | 7.02 | 906 | 27 | 2.26 | 38.82 |
| 582 | Chikkaballapura | 819 | 452 | 64.7 | 4.76 | 819 | 8 | 0.62 | 45.48 |
| 583 | Bangalore rural | 874 | 429 | 58.23 | 17.62 | 874 | 34 | 1.85 | 84.90 |
| 584 | Ramanagara | 766 | 347 | 55.93 | 4.08 | 766 | 13 | 1.18 | 26.37 |
|  | **Goa** |  |  |  |  |  |  |  |  |
| 585 | North goa | 890 | 185 | 20.85 | 58.17 | 890 | 115 | 8.83 | 63.72 |
| 586 | South goa | 806 | 222 | 34.56 | 33.36 | 806 | 79 | 7.98 | 43.11 |
|  | **Lakshadweep** |  |  |  |  |  |  |  |  |
| 587 | Lakshadweep | 1070 | 387 | 29.71 | 45.39 | 1,070 | 75 | 4.92 | 42.54 |
|  | **Kerala** |  |  |  |  |  |  |  |  |
| 588 | Kasaragod | 823 | 361 | 42.61 | 45.76 | 823 | 66 | 3.32 | 73.06 |
| 589 | Kannur | 753 | 340 | 49.24 | 25.03 | 753 | 60 | 3.69 | 46.48 |
| 590 | Wayanad | 909 | 476 | 57.77 | 29.64 | 909 | 80 | 5.44 | 42.70 |
| 591 | Kozhikode | 901 | 483 | 57.46 | 43.6 | 901 | 69 | 3.12 | 66.06 |
| 592 | Malappuram | 992 | 503 | 43.05 | 51.1 | 992 | 103 | 3.1 | 72.81 |
| 593 | Palakkad | 816 | 509 | 62.16 | 40.83 | 816 | 85 | 4.17 | 60.21 |
| 594 | Thrissur | 749 | 436 | 63.84 | 31.64 | 749 | 77 | 4.89 | 54.27 |
| 595 | Ernakulam | 740 | 408 | 57.83 | 46.08 | 740 | 76 | 4.95 | 56.39 |
| 596 | Idukki | 710 | 409 | 63.04 | 41.38 | 710 | 67 | 3 | 62.27 |
| 597 | Kottayam | 704 | 333 | 52.9 | 29.91 | 704 | 90 | 7.79 | 50.44 |
| 598 | Alappuzha | 718 | 335 | 45.09 | 51.19 | 718 | 76 | 6.01 | 64.46 |
| 599 | Pathanamthitta | 642 | 323 | 50.93 | 43.07 | 642 | 80 | 5.39 | 67.46 |
| 600 | Kollam | 793 | 394 | 53.07 | 45.26 | 793 | 85 | 4.44 | 75.64 |
| 601 | Thiruvananthapur | 783 | 370 | 49.09 | 51.73 | 783 | 86 | 5.03 | 72.74 |
|  | **Tamil Nadu** |  |  |  |  |  |  |  |  |
| 602 | Thiruvallur | 911 | 498 | 64.03 | 35.87 | 911 | 70 | 4.03 | 70.25 |
| 603 | Chennai | 928 | 478 | 60.09 | 18.46 | 928 | 56 | 3.39 | 63.88 |
| 604 | Kancheepuram | 958 | 524 | 61.63 | 29.2 | 958 | 67 | 4.18 | 67.85 |
| 605 | Vellore | 954 | 514 | 64 | 26.57 | 954 | 44 | 1.8 | 80.91 |
| 606 | Tiruvannamalai | 916 | 384 | 48.89 | 22.36 | 916 | 36 | 1.67 | 69.96 |
| 607 | Viluppuram | 992 | 433 | 49.93 | 30.83 | 992 | 63 | 2.29 | 56.73 |
| 608 | Salem | 988 | 427 | 52.11 | 18.43 | 988 | 66 | 4.1 | 45.49 |
| 609 | Namakkal | 901 | 449 | 59.35 | 12.98 | 901 | 26 | 1.94 | 36.99 |
| 610 | Erode | 1003 | 528 | 63.01 | 18.28 | 1,003 | 53 | 4.48 | 38.41 |
| 611 | The nilgiris | 973 | 457 | 56.5 | 14.63 | 973 | 24 | 0.82 | 67.16 |
| 612 | Dindigul | 995 | 483 | 60.29 | 8.38 | 995 | 35 | 2.43 | 36.21 |
| 613 | Karur | 973 | 484 | 57.62 | 15.18 | 973 | 85 | 5.73 | 37.83 |
| 614 | Tiruchirappalli | 785 | 332 | 43.43 | 40.36 | 785 | 90 | 5.8 | 55.29 |
| 615 | Perambalur | 912 | 351 | 40.98 | 36.1 | 912 | 92 | 5.37 | 51.88 |
| 616 | Ariyalur | 806 | 245 | 35.92 | 23.23 | 806 | 31 | 3.2 | 45.89 |
| 617 | Cuddalore | 961 | 467 | 55.46 | 28.61 | 961 | 57 | 1.97 | 77.52 |
| 618 | Nagapattinam | 935 | 445 | 57.41 | 28.12 | 935 | 86 | 4.62 | 72.21 |
| 619 | Thiruvarur | 887 | 418 | 55.28 | 23.71 | 887 | 38 | 2.45 | 69.28 |
| 620 | Thanjavur | 868 | 380 | 48.49 | 31.42 | 868 | 75 | 4.92 | 54.69 |
| 621 | Pudukkottai | 912 | 336 | 40.63 | 44.35 | 912 | 76 | 4.99 | 72.12 |
| 622 | Sivaganga | 876 | 317 | 43.34 | 8.66 | 876 | 42 | 2.01 | 60.96 |
| 623 | Madurai | 796 | 364 | 44.47 | 46.38 | 796 | 97 | 4.65 | 71.26 |
| 624 | Theni | 792 | 294 | 38.5 | 28.75 | 792 | 46 | 2.84 | 53.51 |
| 625 | Virudhunagar | 738 | 161 | 23.33 | 33.66 | 738 | 38 | 2.71 | 72.23 |
| 626 | Ramanathapuram | 777 | 186 | 25.98 | 32.9 | 777 | 44 | 2.06 | 85.94 |
| 627 | Thoothukkudi | 761 | 201 | 30.07 | 26.21 | 761 | 39 | 2.53 | 68.85 |
| 628 | Tirunelveli | 709 | 215 | 36.07 | 25.94 | 709 | 35 | 2.51 | 54.07 |
| 629 | Kanniyakumari | 836 | 323 | 45.05 | 27.49 | 836 | 41 | 2.33 | 69.49 |
| 630 | Dharmapuri | 965 | 437 | 54.98 | 15.29 | 965 | 34 | 2.11 | 48.21 |
| 631 | Krishnagiri | 1120 | 562 | 60.77 | 27.71 | 1,120 | 41 | 1.44 | 78.13 |
| 632 | Coimbatore | 1009 | 540 | 65.38 | 16.26 | 1,009 | 51 | 2.79 | 52.55 |
| 633 | Tiruppur | 883 | 478 | 63.08 | 18.59 | 883 | 46 | 2.1 | 61.29 |
|  | **Puducherry** |  |  |  |  |  |  |  |  |
| 634 | Yanam | 1013 | 567 | 70.56 | 7.12 | 1,013 | 18 | 1.1 | 56.58 |
| 635 | Puducherry | 1019 | 544 | 65.02 | 14.76 | 1,019 | 62 | 3.63 | 43.52 |
| 636 | Mahe | 1058 | 375 | 40.9 | 26.17 | 1,058 | 45 | 1.72 | 74.50 |
| 637 | Karaikal | 922 | 383 | 48.67 | 30.17 | 922 | 79 | 5.89 | 57.16 |
|  | Andaman & Nicobar islands |  |  |  |  |  |  |  |  |
| 638 | Nicobars | 1019 | 252 | 38 | 6.32 | 1,019 | 26 | 2.23 | 44.83 |
| 639 | North & middle Andaman | 842 | 465 | 66.87 | 18.32 | 842 | 114 | 12 | 30.45 |
| 640 | South Andaman | 950 | 390 | 45.56 | 56.66 | 950 | 110 | 7.61 | 56.16 |

**Appendix S2: Result of Bivariate relationship between high modern spacing contraceptive prevalence rate with low discontinuation of modern spacing method and low modern spacing contraceptive prevalence rate with high discontinuation of modern spacing method from LISA cluster map, 2015-16**

| **Districts (Low-High)** | **Districts (High-Low)** |
| --- | --- |
| **Andhra Pradesh** | **Himachal Pradesh** |
| Nalgonda | Sirmaur |
| Warangal | **Uttar Pradesh** |
| Nizamabad | Saharanpur |
| Medak | Ghaziabad |
| Rangared | **Haryana** |
| Mahbubnagar | Kurukshetra |
| Kurnool | Karnal |
| Prakasam | Hisar |
| Chittoor | **Maharashtra** |
| Visakhapatnam | Nagpur |
| **Karnataka** | Amaravati |
| Bellary | Akola |
| Davanagere | Buldana |
| Chitradurga | Washim |
| Shimoga | **Odisha** |
| Chikmagalur | Nayagarh |
| Dakshina Kannada | Subarnapur |
| Kodagu | **West Bengal** |
| Bangalore | Puruliya |
| Ramanagara | Barddhaman |
| **Uttarakhand** | North 24 |
| Uttarakhand | Hugli |
| **Kerala** | Darjeeling |
| Wayanad | **Sikkim** |
| Thrissur | North |
| Palakkad | West |
| Idukki | **Assam** |
| Kottayam | Baksa |
| Alappuzha | Kamrup |
| Pathanamthitta | Marigaon |
| **Tamil Nadu** | **Meghalaya** |
| Theni | Ri Bhoi |
| Tirunelveli | **Mizoram** |
| Thoothukkudi | Mamit |
| Virudunagar | **Tripura** |
| Pudukkottai | North Tripura |
| Thanjavur | Dhalai |
| Nagappattinam | South Tripura |
| Viluppuram | West Tripura |
| Kanchipuram | **Nagaland** |
| **Uttar Pradesh** | Zunheboto |
| Maharajganj | Phek |
| Siddharth Nagar | Tuensanga |
| Balrampur | **Arunachala Pradesh** |
| Shrawasti | Changlang |
| Gonda | Anjaw |
| Gorakhpur | WestKameng |
| Kushinagar | Sonitpur |
| **Bihar** |  |
| Paschim champaran |  |
| Sitamarhi |  |
| Purba Champaran |  |
| Darbhanga |  |
| Supaul |  |
| Saran |  |

**Appendix S3: Estimated result of spatial weighted OLS regression model for any modern spacing contraceptive method, 2015-16**

| **Variable** | **Coefficient** | **P-value** |
| --- | --- | --- |
|  |  |  |
| Constant | 17.99 | 0.00 |
| Mean year schooling | 2.33 | 0.10 |
| Method Information Index | -0.001 | 0.97 |
| Parity 2+ | -0.35 | 0.10 |
| Unmet need | 0.88 | 0.00 |
| Female sterilization | 0.60 | 0.00 |
| Method attribute and failure opposition | 0.05 | 0.15 |
| Desire for children | 0.24 | 0.11 |
| Visited health facility by any health issue | -0.01 | 0.80 |
| Schedule Caste/Schedule Tribe | -0.12 | 0.00 |
| Urban | -0.08 | 0.00 |
| Occupation | 0.12 | 0.05 |
| Number of districts | 640 | |
| R^2^ | 0.15 | |
| Adjusted R^2^ | 0.13 | |
| AIC | 5237 | |
| Log likelihood | -2606 | |
| **Spatial dependency test** | **MI/DF** | **P-value** |
| Moran's I (error) | 0.21 | 0.00 |
| Lagrange Multiplier (lag) | 1 | 0.00 |
| Robust LM (lag) | 1 | 0.05 |
| Lagrange Multiplier (error) | 1 | 0.00 |
| Robust LM (error) | 1 | 0.01 |
| Lagrange Multiplier (SARMA) | 2 | 0.00 |

**Appendix S4: Classification of states by region in India, NFHS-4(2015-16)**

| North | Chandigarh, Delhi, Haryana, Himachal Pradesh, Jammu & Kashmir, Punjab, Rajasthan, Uttarakhand |
| --- | --- |
| Central | Chhattisgarh, Madhya Pradesh, Uttar Pradesh |
| East | Bihar, Jharkhand, Odisha, West Bengal |
| North East | Arunachal Pradesh, Assam, Manipur, Meghalaya, Mizoram, Nagaland, Sikkim, Tripura |
| West | Dadra & Nagar Haveli, Daman & Diu, Goa, Gujarat, Maharashtra |
| South | Andaman & Nicobar, Islands, Andhra Pradesh, Karnataka, Kerala, Lakshadweep, Puducherry, Tamil Nadu, Telangana |
